# The quantitative amino acid economy of yeast reveals public versus private goods and guides syntrophic community design

**DOI:** 10.1128/msystems.00333-26

**Published:** 2026-08-07

**Authors:** Shabbir Ahmad, Ganesh Muthu, Sunil Laxman

**Affiliations:** 1 Institute for Stem Cell Science and Regenerative Medicine (BRIC inStem), Bangalore, India; 2 Manipal Academy for Higher Education (MAHE), Manipal, India

**Keywords:** amino acid economy, metabolic flux, *S. cerevisiae*, auxotrophy, synthetic communities

## Abstract

The biosynthetic capacity of a cell governs the production and exchange of amino acids. Given the distinct metabolic origins and intracellular requirements for amino acids, it is essential to establish quantitative amounts of internal amino acid pools and how these change across growth phases. Using *Saccharomyces cerevisiae*, here we establish an absolute, quantitative blueprint of the intracellular and extracellular amino acid economy, defining the fluxes of production, secretion, and consumption across 24 hours of cell growth. While the intracellular pool is dominated by a group of amino acids, their relative proportions continuously change over time. The extracellular pool is notably distinct in terms of composition and amounts. Only select amino acids are public goods secreted in significant amounts, and a subset of these (Ala, Val, Gln, Trp, and Phe) are subsequently re-consumed. Five amino acids, Asp, Lys, His, Arg, and Met, remain “privatized” even in nutrient abundance. We demonstrate that the strictly privatized amino acid Asp continuously sustains diverse carbon metabolism, while the public Ala is utilized following carbon depletion. Furthermore, nitrogen limitation triggers a significant shift toward storage, privatizing otherwise abundant nitrogen-rich compounds like Gln, Asn, and Pro. We thereby rationally establish pairs of stable synthetic communities of paired public good auxotrophs that show effective growth. Our results identify frameworks for feasible amino acid trade and provide a basis for engineering stable, synthetic communities of amino acid auxotrophs.

Cells substantially produce, utilize, and accumulate metabolic resources such as amino acids, and these resources can be secreted outside to be traded between cells. Through such trade, cells assemble into syntrophic communities—commonly seen in microbial systems (1–3), or even within tissues. This metabolic exchange between cells allows distributed metabolic functions between cell groups, divided metabolic labor, increased metabolic productivity, or reduced biosynthetic load (1, 3–5). All these suggest that by rationally manipulating these amino acid fluxes, it may be possible to engineer cells to assemble as exchange-based synthetic communities or sustain complex biosynthesis based on precursor supply. Such efficiencies due to exchange are seen in auxotrophic microbial syntrophic consortia (3, 6–13), and even in extreme examples in nature such as aphid insect-bacterial endosymbionts, which survive based on extensive amino acid trade and associated metabolic optimizations (14, 15). Metabolic trade can even occur within clonal colonies of prototrophic cells (which can biosynthesize their own metabolites), through the self-organization into cell groups with division of labor to share resources (1, 16, 17). Thus, biochemical and thermodynamic constraints clearly drive the assembly of cell communities (3, 18, 19). However, specific rules that determine which metabolic resources are exchanged between cells and how much cells prioritize storage vs. exchange of resources such as amino acids remain poorly understood.

Amino acids are commonly exchanged between microbial cells (5, 20–23); yet, they are not homogeneous but are chemically, metabolically, and functionally distinct and present at different amounts (24, 25). Cells maintain different extents of strategic amino acid reserves for metabolic and translational requirements (26). Consequently, cells function as demand-driven economies with respect to different amino acids, where the net demand for each amino acid shapes hierarchies of resource allocations (24). In order for an amino acid to be traded, it must be available in excess of demand—which is a balance governed by its cycles of production, consumption, and secretion. Therefore, a first principles understanding of the cellular amino acid economy requires absolute, temporal quantifications of both intracellular and extracellular pools across cell growth phases. This knowledge can also aid in designing biosynthetic processes in engineered cells to produce amino acid–derived molecules. However, there is limited quantitative information available for temporal changes in the amino acid economy, even for model microbes. Our current quantitative knowledge is limited to snapshots from *Escherichia coli* or yeast in the “log phase” growth (25, 27, 28), and the quantitative dynamics of amino acid production, secretion, and temporal pool changes are largely uncharacterized.

In this study, we utilized the eukaryotic model cell *Saccharomyces cerevisiae*, which is prototrophic for the biosynthesis of all amino acids, to quantitatively determine the absolute concentrations of intracellular and extracellular amino acids and biosynthetic flux rates across a 24-hour growth phase in defined glucose- and ammonium-replete medium. We show that cells maintain distinct amounts and magnitudes of intracellular and extracellular amino acids that do not correlate with each other in abundance, demand, or hierarchies of utilization. Specific amino acids are privatized, secreted minimally to the external medium, and continuously support critical intracellular metabolic processes. Others are secreted as public goods into the extracellular environment, a subset of which are taken up and re-consumed to support metabolism of the producing cell. Under nitrogen limitation, cells shift toward significant privatization, particularly of nitrogen-rich amino acids. Using this quantitative blueprint of public and private goods amino acids, we rationally designed synthetic consortia by pairing auxotrophs for specific “public good” amino acids. These established stable, syntrophic communities that grew robustly together. Our data reveal quantitative hierarchies of cellular amino acid allocation, with implications for the design of synthetic consortia for metabolic engineering applications.

## Results

### Amino acid biosynthetic flux peaks during early growth and is decoupled from gene expression

**Fig 1:**
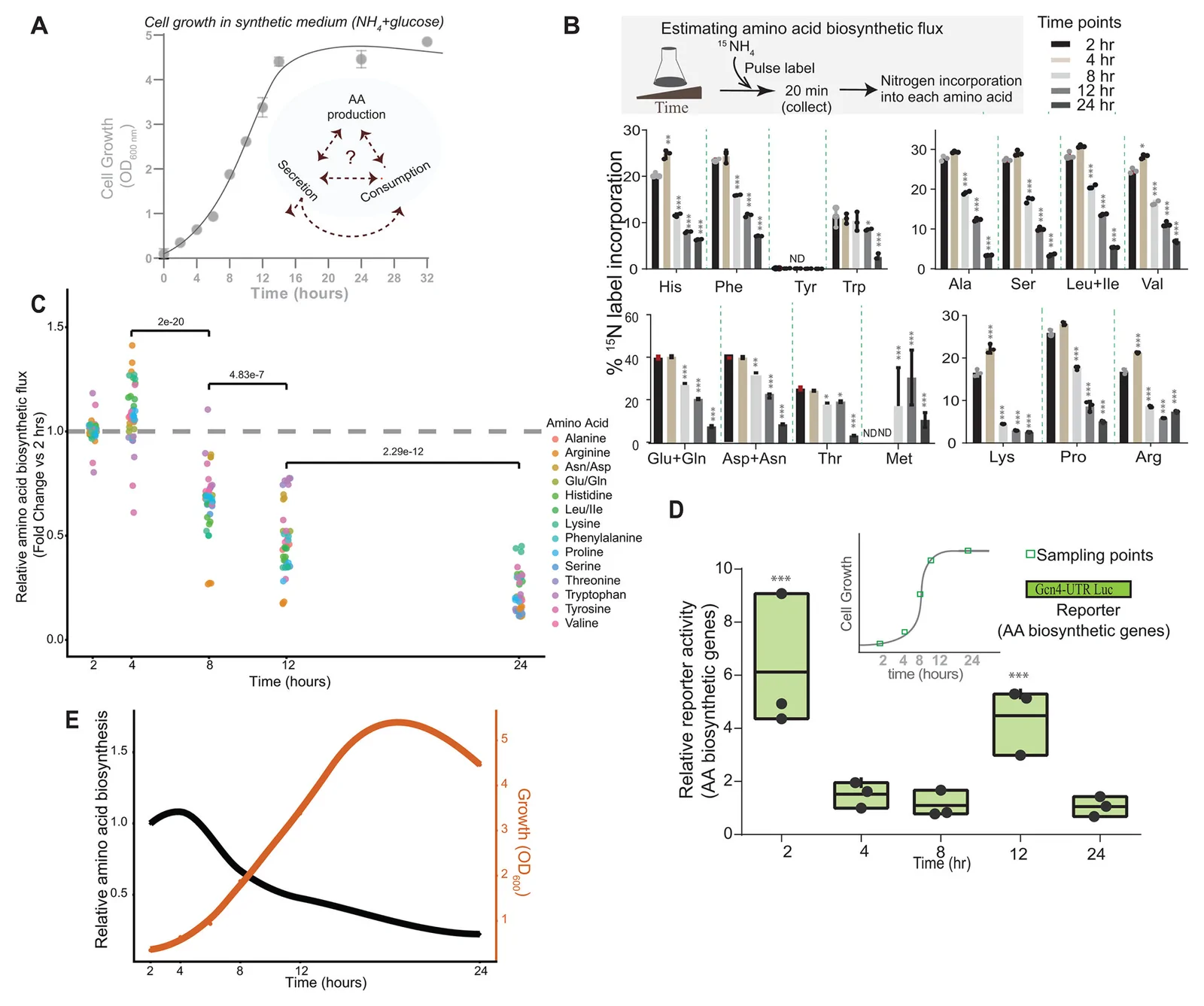
Amino acid biosynthetic flux peaks during early growth and is decoupled from gene expression. (A) Cells modulate and balance amino acid production, secretion, and consumption during different growth phases, but the dynamics and interplay of this production, secretion, and consumption are poorly understood. On the right is a standard growth curve of *S. cerevisiae* in batch culture over ~ 24 hours in synthetic, defined medium with glucose and ammonium sulfate as the sole carbon and nitrogen sources, respectively (*n* = 3, mean ± SD), also indicating sampling points where subsequent amino acid analyses were performed. (B) Estimates of relative amino acid biosynthetic flux in cells after 2, 4, 8, 12, and 24 hours of growth in synthetic medium. (Upper inset) Experimental workflow used to estimate amino acid biosynthetic flux, using a pulse-label of ^15^N labeled ammonium sulfate and measuring label incorporation into the respective amino acid using targeted liquid chromatography–tandem mass spectrometry (LC-MS/MS), from cells collected at the indicated time points. The relative label incorporation into each newly synthesized amino acid is shown. The amino acids are presented as groups loosely based on their metabolic origins. Data are from three biological replicates (*n* = 3). Comparisons are to the 2-hour time point of each sample. Significance by Student’s *t*-test. *P < 0.05, **P < 0.01, ***P < 0.001. ND, not detected. (C) Normalized relative biosynthetic flux for all amino acids. The scatter plot shows relative biosynthetic flux for all amino acids, tracked over 24 hours. Each time point for every amino acid is normalized to the 2-hour time point (baseline indicated by the horizontal dashed gray line). Statistical differences between consecutive time intervals are evaluated using two-sample *t*-tests. (D) Transcriptional induction of amino acid biosynthetic genes across 24 hours of growth. The extent of transcriptional activation of amino acid biosynthetic genes in distinct phases of growth was estimated using the activity of an established transcriptional reporter for amino acid biosynthesis (Gcn4-luc) (24). Cells were grown in synthetic minimal medium, as indicated in panel A. Normalized relative reporter activity is measured (*n* = 3, mean ± SD). (E) Summary of biomass vs. amino acid biosynthesis over 24 hours. Cells continue to grow in synthetic, defined medium, reaching the stationary phase between 12 and 24 hours. In contrast, amino acid biosynthetic flux is highest after ~4 hours of growth, and decreases substantially by 12 hours.

The amounts of intracellular amino acids reflect a steady state coming from the net amount of production, consumption, and secretion of amino acids. How do the cycles of amino acid production, consumption, and secretion change temporally as cells transition from exponential growth to the stationary phase? (Fig. 1A). Quantitative estimates of these dynamic amino acid fluxes are missing even in well-studied model microbial systems (Fig. 1A). To address this, we used prototrophic yeast cells grown in defined minimal medium without supplemented amino acids. In this study, we used a robust, prototrophic yeast strain (CEN.PK background) (29) grown in batch culture in synthetic, defined minimal medium with 110 mM glucose and ~35 mM ammonium sulfate as sole carbon and nitrogen sources respectively (SD). In this condition, prototrophic yeast carry out *de novo* biosynthesis of all amino acids, thereby allowing a quantitative assessment of amino acids over time, across phases of growth.

We first determined the changes in amino acid biosynthetic flux during 24 hours of cell growth, to identify the phase of growth where cells maximize amino acid biosynthesis. To quantify the relative rate of amino acid biosynthetic flux over this 24-hour window, we designed a metabolic flux experiment, using a short pulse of stable-isotope-labeled nitrogen precursors (^15^N-labeled ammonium sulfate), followed by assessing the relative nitrogen label incorporation into newly synthesized amino acids. ^15^N-labeled ammonium sulfate was pulsed briefly into the culture at the indicated time points (viz. 2, 4, 8, 12, and 24 hours), cells were collected, and the incorporation of ^15^N-label into newly synthesized amino acids was estimated by quantitative, targeted LC-MS/MS (Fig. 1B). We observed the highest amino acid biosynthetic flux during the first 2–4 hours of cell growth (Fig. 1B). This declines substantially and by 24 hours, when cells have entered the stationary phase, biosynthesis reached a minimum (Fig. 1B). Notably, this temporal trend was significantly consistent for all amino acids and was not specific or restricted to a single group of amino acids (Fig. 1C), establishing a clear downregulation of all amino acid biosynthetic activities. These data establish that most amino acid biosynthesis is completed in the early exponential phase of growth.

We next asked whether amino acid biosynthetic gene expression accurately reflected actual biosynthetic flux across growth phases. While gene expression is frequently used as a proxy for metabolic activity, it may fail to distinguish between active biosynthesis and starvation-induced transcriptional responses (27, 30, 31). To assess the temporal expression of amino acid biosynthetic genes, we used a well-established luciferase-based reporter for amino acid biosynthetic gene expression (24, 32), based on the activity of the amino acid master-regulating transcription factor Gcn4 (32–34) (Fig. 1D). Reporter activity was measured at 2, 4, 8, 12, and 24 hours (corresponding to the flux measurements). The reporter activity revealed two distinct waves of expression of amino acid biosynthetic genes (Fig. 1D). The first peak coincided with maximum biosynthetic flux during early growth phases (2–4 hours). A second peak of activity followed at ~12 hours (Fig. 1D), when actual biosynthetic flux had already reached a minimum. These results demonstrate a separation between transcription and metabolism in later growth phases, and the second wave of transcription likely represents a starvation response, where cells sense reductions in actual amino acid biosynthetic flux as nutrients deplete, despite the lack of precursors or energy required to sustain actual flux.

Collectively, these data comprehensively establish the temporal dynamics of amino acid production, where the majority of biosynthesis is completed during early phases of growth (Fig. 1E).

### Intracellular and extracellular amino acid hierarchies are distinct, dynamic and metabolically uncoupled

We next asked what the absolute intracellular and extracellular amino acid concentrations in yeast cells are over the 24-hour growth cycle. It remains unknown what these concentrations are, how these concentrations differ between distinct amino acids or how the internal pool relates to the external pool (of secreted amino acids). Absolute quantification is necessary to determine the balance between intracellular demand and production of different amino acids, and to identify which amino acids are effectively shared publicly into the external environment.

**Fig 2:**
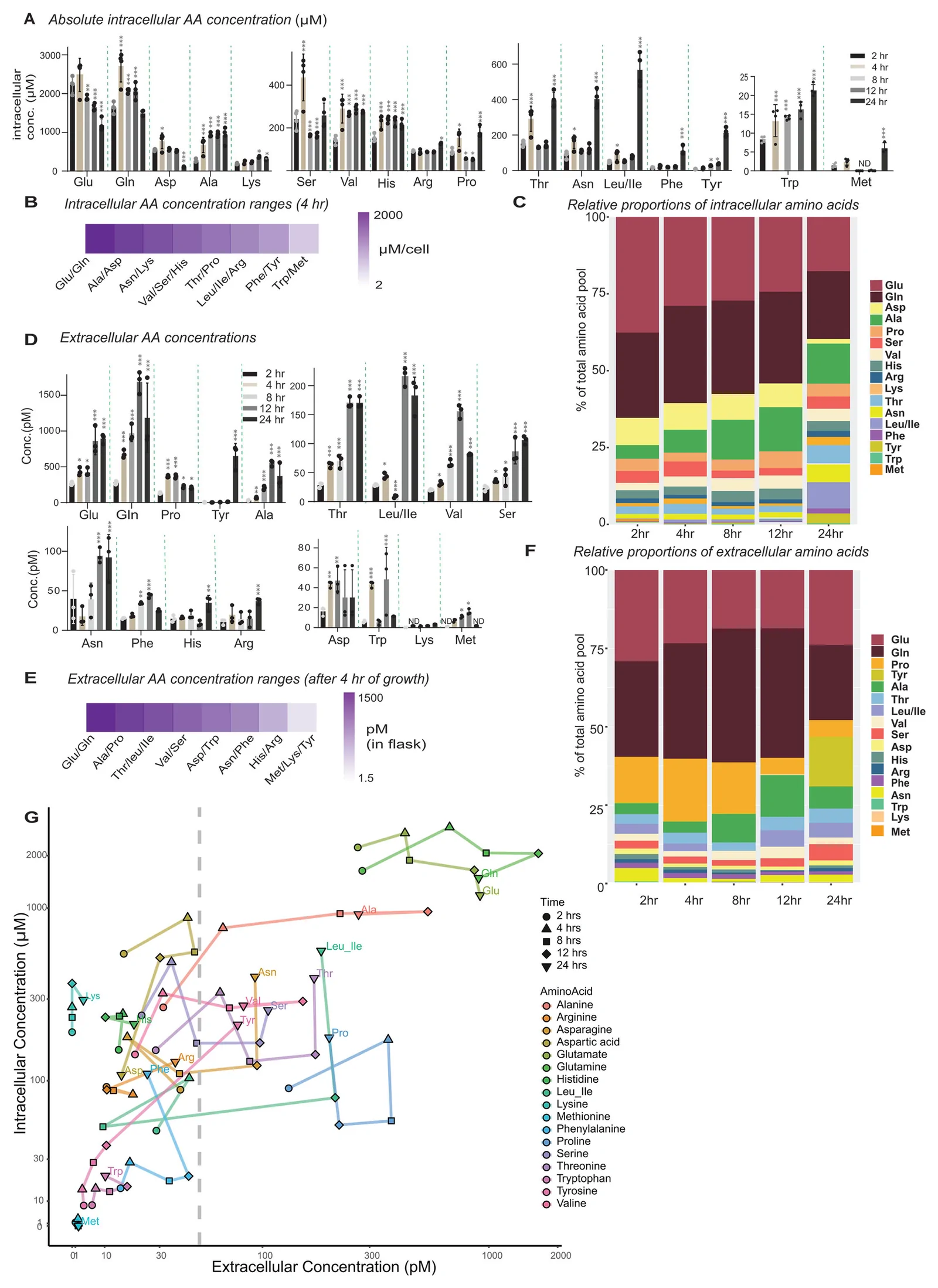
Intracellular and extracellular amino acid hierarchies are distinct, dynamic, and metabolically uncoupled. (A) Absolute concentrations of intracellular amino acids from cells collected at the indicated growth phases. Intracellular amino acids were quantitatively estimated by LC-MS/MS. The amino acids are arranged based on the order of their intracellular concentration (high/intermediate/low in μM/cell). The estimated volume of a single yeast cell is 40 fl. *n* = 4 (biological replicates). Significance comparisons are to the 2-hour time point of each sample (Student’s *t*-test). *P < 0.05, **P < 0.01, ***P < 0.001. (B) A heatmap to illustrate relative abundances of groups of amino acids inside a cell after 4 hours of growth. The purple color intensity reflects the amount of amino acid present, over three orders of magnitude (μM/cell). (C) Proportions of intracellular amino acids over time at different cell growth phases: Stacked bar plots illustrate changes in the relative proportions of distinct intracellular amino acids across various phases of growth. Note that the relative proportions of several amino acids change substantially at different times. Also see Fig. S1. (D) Absolute concentrations of extracellular amino acids (accumulated in the medium) across the indicated times of growth. Measurements were made from the extracellular medium supernatants, and all experiments were carried out using 30 mL of culture medium in shake flasks. The extracellular amino acids are arranged from the most to least abundant at the indicated time points. Note that while glutamate and glutamine remain the most abundant extracellular amino acids, several other amino acids in the extracellular environment are present at amounts that do not correlate with their intracellular amounts (panel 1B). Some amino acids are not detectable or present at very small amounts in the extracellular environment. n = 3, mean ± SD (biological replicates). Significance comparisons are to the 2-hour time point of each sample (Student’s *t*-test). *P < 0.05, **P < 0.01, ***P < 0.001. (E) A heatmap to illustrate relative abundances of groups of amino acids outside a cell in the extracellular medium after 4 hours of growth. The purple color intensity reflects the amount of amino acid present, over three orders of magnitude. The concentrations are in pM. (F) Proportions of extracellular amino acids over time across growth phases: Stacked bar plots illustrate changes in the relative proportions of distinct intracellular amino acids across various phases of growth. Note that the relative proportions of several amino acids change substantially at different times. Also see Fig. S2. (G) A scatter plot, visualized on a pseudo-log scale, representing absolute intracellular concentrations (μM/cell) versus extracellular amino acid concentrations (pM), over 24 hours of growth. Only a subset of amino acids are ever present in the extracellular medium at over 50 pM. Some amino acids (lys and met) are present at extremely low/undetectable amounts in the extracellular medium at all measured time points.

We therefore used highly quantitative, targeted LC-MS/MS approaches (35) to determine absolute intracellular amino acid amounts at 2, 4, 8, 12, and 24 hours of growth. Concentrations were calculated based on cell number and volume (Fig. 2A and Table 1; see Materials and Methods). Our data reveal substantial differences in amounts of different amino acids, with concentrations spanning ~4 orders of magnitude (Fig. 2A). Consistent with studies from bacteria (25) and recent predictions (24), the most abundant amino acids were glutamate, glutamine, alanine, and aspartic acid—which were maintained concentrations in high micromolar to millimolar amounts within the cell, while methionine was among the lowest across different phases of growth (Fig. 2A and B and Table 1). Note: glycine and cysteine were not quantified due to technical reasons or redox sensitivity, respectively; however, our earlier studies establish cysteine concentrations in the high nM range (36). The relative amounts of intracellular amino acids at 4 hours—representing the peak of biosynthetic flux—are visualized in Fig. 2B.

### Shifts in proportional intracellular composition

Over this temporal window, the absolute intracellular concentrations of amino acids, as well as relative proportions of each amino acid as a fraction of total amino acids inside a cell change markedly (Fig. 2C and Table 1). Glutamate and glutamine remain the most abundant intracellular amino acids across this time window, but their intracellular amounts decrease steadily. Some amino acids, notably aspartate, declined across all growth phases, while a distinct group of amino acids including histidine, lysine, and the branched-chain amino acids (BCAAs) accumulated within the cell (Fig. 2C). Notably, intracellular arginine levels were maintained over time (Fig. 2A and C and Table 1). Following the 12-hour mark, we observed a substantial proportional increase in aromatic amino acids (Phe, Tyr, and Trp), BCAAs (Val and Leu/Ile) and asparagine (Fig. 2C and Table 1).

These results suggest a dynamic internal amino acid economy characterized by two features: (i) some amino acids are likely produced in excess beyond cellular demand and (ii) the relative proportions of intracellular pools are not static, but shift according to the growth state of the cell (Fig. 2C). These internal dynamics raise the question, how the composition of the extracellular amino acid pool changes in this same temporal window. Which amino acids are secreted substantially by cells, and are there distinct cycles of re-consumption? Furthermore, are certain amino acids strictly retained within the cell?

### Extracellular amino acids

We therefore next quantified absolute amounts of extracellular amino acids from the culture supernatant over the 24-hour period. Samples were collected from the extracellular medium (with cells removed), and concentrations were determined via targeted LC-MS/MS (Table 1), and concentrations were calculated as picomolar amounts present in this extracellular medium. Note that all experiments were done in 30 mL of liquid culture volume, from which molar concentrations were calculated appropriately. Extracellular amino acid profiles arranged by abundance, are shown in Fig. 2D. Notably, only a subset of all the amino acids was easily detectable in the extracellular medium (Fig. 2D). Similar to intracellular trends, glutamine and glutamate were the most abundant, followed by alanine. Furthermore, proline, branched-chain amino acids, and threonine were present in substantial amounts in the extracellular medium (Fig. 2D and E and Table 1). In addition, serine, asparagine, threonine, and leucine/isoleucine were also substantially present in the extracellular environment (Fig. 2D), also consistent with their intracellular accumulation patterns (Fig. 2A through C). Additionally, histidine and arginine displayed an increase in extracellular concentrations, from 12 to 40 pM (from the culture supernatant) between 12 and 24 hours (Fig. 2D and E). At these times, these amino acids were also high within the cell, with absolute intracellular concentrations of 80 to ~250 µM (Fig. 2A). Cells therefore secrete significant amounts of select amino acids during exponential growth, when nutrient availability is significant amounts (Fig. 2D and E).

Notably, a subset of amino acids, primarily histidine, arginine, aspartic acid, methionine, and lysine, remained at near detection limits (10–40 pM) or were undetectable in the extracellular medium (Fig. 2D and E and Table 1). These consolidated, temporal dynamics of intracellular and extracellular amino acid pools are depicted together in the scatter graphs shown in Fig. 2G. Two primary conclusions clearly emerge. First, the extracellular amino acid environment is not static but changes in composition throughout culture growth (Fig. 2F and G). Second, the decline in select extracellular amino acid amounts over time suggests phases where there is subsequent reuptake (Fig. 2F and Table 1). Given the divergent temporal dynamics of intracellular and extracellular amino acid pools, we conclude that the extracellular environment does not reflect the intracellular metabolic state—either in absolute amounts or in relative proportions. Importantly, some amino acids (lysine, aspartic acid, arginine, and histidine), which are abundant inside the cell, are almost unavailable outside.

Collectively, these results demonstrate a divergence between intracellular and extracellular amino acid pools, where a subset of amino acids are retained within (Fig. 2G). Additionally, there is no obviously inferred relationship between the biosynthetic cost of an amino acid and its extracellular availability. Many abundantly secreted amino acids have high biosynthetic costs (proline, the branched-chain amino acids, and threonine); however, many others have very low biosynthetic costs in these media conditions (glutamine, glutamate, alanine, etc.) (24). All the amino acids excreted, however, are made in amounts exceeding their demand, and this is further discussed later in the manuscript. These data are largely consistent with an overall demand-driven cellular amino acid economy, as established earlier (24). This is explored in more detail in the next section.

### Distinct amino acids function as private, consumed, or surplus public goods

**Fig 3:**
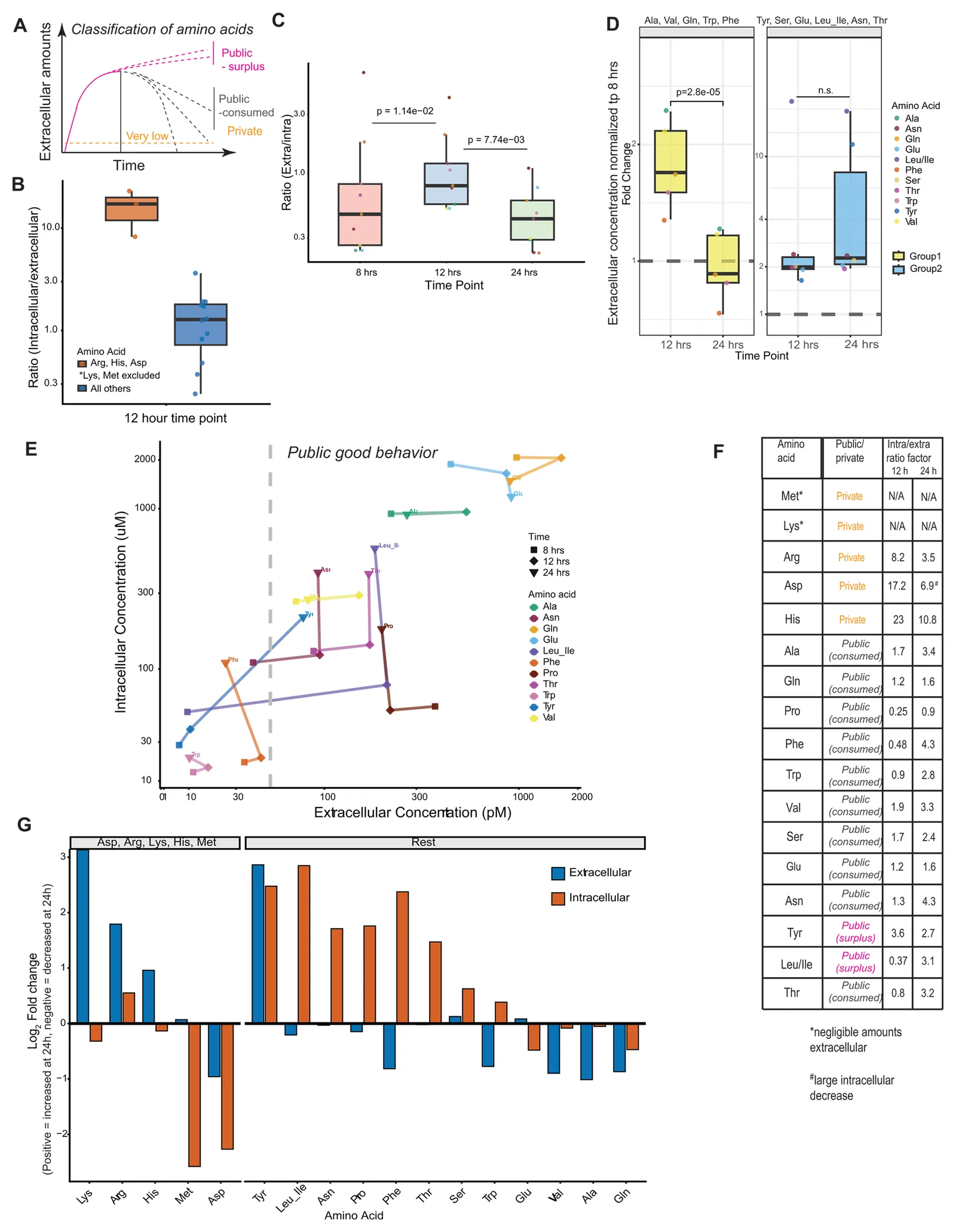
Distinct amino acids function as private, consumed, or surplus public goods. (A) Classification of amino acids as private or public goods based on the cut-offs defined in Fig. 2G comparing intracellular and extracellular amino acid amounts. Amino acids can be classified as private goods or two types of public goods (consumed or surplus) based on extracellular availability over time. Privatized amino acids can be conservatively defined as those that are never present in significant amounts in the extracellular culture medium. Public good amino acids would all be present at significant amounts (at some time) in the extracellular medium. Consumed public goods are those that initially accumulate in the extracellular medium and subsequently decrease in abundance (defined as those that increased at 24 hours, over 12 hours). Surplus public goods will continuously accumulate in the extracellular culture medium over time (defined as increased between 12 and 24 hours). (B) Privatized amino acids. Two amino acids—Met and Lys—were present at very low amounts/not detected in the extracellular medium. Three additional amino acids, Asp, His, and Arg, were also present at very low extracellular amounts of <50 pM (in the medium supernatant) at any time. For intracellular and extracellular amino acids at 12 hours of growth, we devised an intra./extra. ratio factor (also see panel G), and the ratio factor of Asp His, and Arg compared with that of all other amino acids is shown. (C) Public amino acids. All other amino acids (excluding Met, Lys, Asp, Arg, and His) are public, and collectively increase significantly in the extracellular medium after 12 hours (paired *t*-test, *P* = 0.0114). Collectively, these public amino acids also decrease significantly after 24 hours (paired *t*-test, *P* = 0.0077). (D) Consumed public good amino acids. Five amino acids, Ala, Gln, Val, Trp, and Phe, which are all present at substantial amounts in the extracellular medium, decrease significantly (paired *t*-test, *P* = 2.8 × 10^−5^) at 24 hours, compared to 12 hours, indicating update and subsequent utilization or storage by cells. In comparison, other public amino acids—Tyr, Ser, Glu, Leu/Ile, Asn, and Thr—do not show any significant decrease after 24 hours in the extracellular medium, indicating that these are produced (and secreted) at substantial surplus by cells. (E) The scatter plots indicate collective trends for intracellular and extracellular amounts for the public amino acids between 8 and 24 hours of growth. After 24 hours, alongside the decrease in extracellular amounts observed in 3D for select public goods, an increase in intracellular amounts can also be observed. (F) A consolidated classification of all amino acids, with an intracellular/extracellular ratio factor included. At 12 hours, when total amino acid amounts (intra. and extra) are cumulatively highest, the private amino acids (Lys, Met, Asp, His, and Arg) are either undetectable in the extracellular medium or have a ratio factor >8. At 24 hours, this ratio largely remains; however, it decreases for Arg, indicating reduced privatization of this amino acid. The ratio factor for all public amino acids remains below 4 at all times. (G) A grouped bar plot displaying the log2 fold change (Log2FC) of absolute intracellular and extracellular concentrations for individual amino acids between 12 and 24 hours of growth. A positive Log2FC value indicates accumulation within that specific compartment at 24 hours relative to 12 hours, whereas a negative value indicates depletion.

Based on the absolute quantification of intracellular and extracellular pools, can amino acids be categorized into distinct economic classes of public and private goods? Public goods are resources that are available to all members of a population, while private goods are exclusively restricted to the producing individuals. For context in bacterial biofilms, a fraction of cells secrete extracellular polymeric substances (EPS), which provide structural stability and protection to the whole community (37). Publicly secreted metabolites also meet this definition, and in some cases may also not be taken back up. In contrast, any amino acid that is not found in any significant amounts extracellularly would practically be a private good. For our initial analyses, we used the data from the 12-hour time window, where cell growth was complete, and where amino acid amounts were the highest levels. The distinct temporal trends observed across amino acids suggest that they do not form a homogeneous metabolic pool. Instead, they can be categorized into two broad groups based on their extracellular availability: (i) private goods and (ii) public goods, the latter of which can be further subdivided into “public goods—consumed” and “public goods—surplus” (Fig. 3A).

We analyzed the 12-hour growth data, representing the peak of amino acid production to define these classes. Public goods were defined as metabolites accessible to the population, while private goods were defined as those strictly retained by the producing cell. We classified amino acids as “private” using two criteria. First, these were either undetectable in the extracellular medium or present at absolute concentrations below 50 pM (in the given extracellular medium supernatants) at any time (Table 1). Additionally, we calculated a “ratio factor” (intracellular/extracellular) to assess the degree of retention. Five amino acids—lysine, methionine, arginine, aspartate, and histidine—clearly met the criteria for private goods at 12 hours. While lysine and methionine were undetectable externally, arginine, histidine, and aspartate maintained higher ratio factors compared to all other detected amino acids (Fig. 3B), confirming their privatization. These data establish that several amino acids maintained at high intracellular concentrations are effectively privatized by the cell.

The remaining amino acids were classified as “public goods.” To determine whether these may be subsequently utilized, we calculated the inverse ratio factor (extracellular/intracellular) at 12 and 24 hours, respectively (Fig. 3C). Notably, this inverse ratio factor decreased at 24 hours compared to 12 hours (Fig. 3C), suggesting overall subsequent consumption. For a more quantitative assessment, we further partitioned these into two categories based on their extracellular depletion at 24 hours compared to 12 hours (Fig. 3D). Ala, Val, Gln, Trp, and Phe showed a significant decrease in extracellular amounts between 12 and 24 hours, classifying them as public goods—consumed. In contrast, Asn, Ser, Thr, Leu/Ile, Tyr, and Glu showed no significant decrease, classifying them as public goods—surplus (Fig. 3D).

The collective dynamics of absolute intracellular and extracellular pools of the public amino acids are visualized as a temporal scatter plot (for 8–24 hours), reflecting the partitioning of these amino acids across the later phases of growth (Fig. 3E and F). Additionally, by 24 hours as cells enter the stationary phase with starvation, we see that the pool of publicly available amino acids changes. We compared the relative changes in intracellular and extracellular amounts between 12 and 24 hours (Fig. 3G) and found that many “public goods—consumed” species increased intracellularly while depleting extracellularly, confirming active uptake and utilization. Interestingly, the strict privatization of Lys, Arg, and His appeared to relax during this phase, as these amino acids became detectable or increased in the extracellular medium. In contrast, aspartate remained uniquely sequestered; it was the only amino acid to show a continuous intracellular decline while its already minimal extracellular levels decreased further (Fig. 3G). Finally, considerable amounts of leucine/isoleucine as well as tyrosine now become publicly available in the extracellular medium at 24 hours (Fig. 3E and G). These data demonstrate that yeast cells dynamically manage amino acid pools through privatization, surplus secretion, or cyclical re-consumption, which are tuned to the metabolic state of the population (Fig. 3F and G).

### Functional allocation of a private and a public amino acid resource

**Fig 4:**
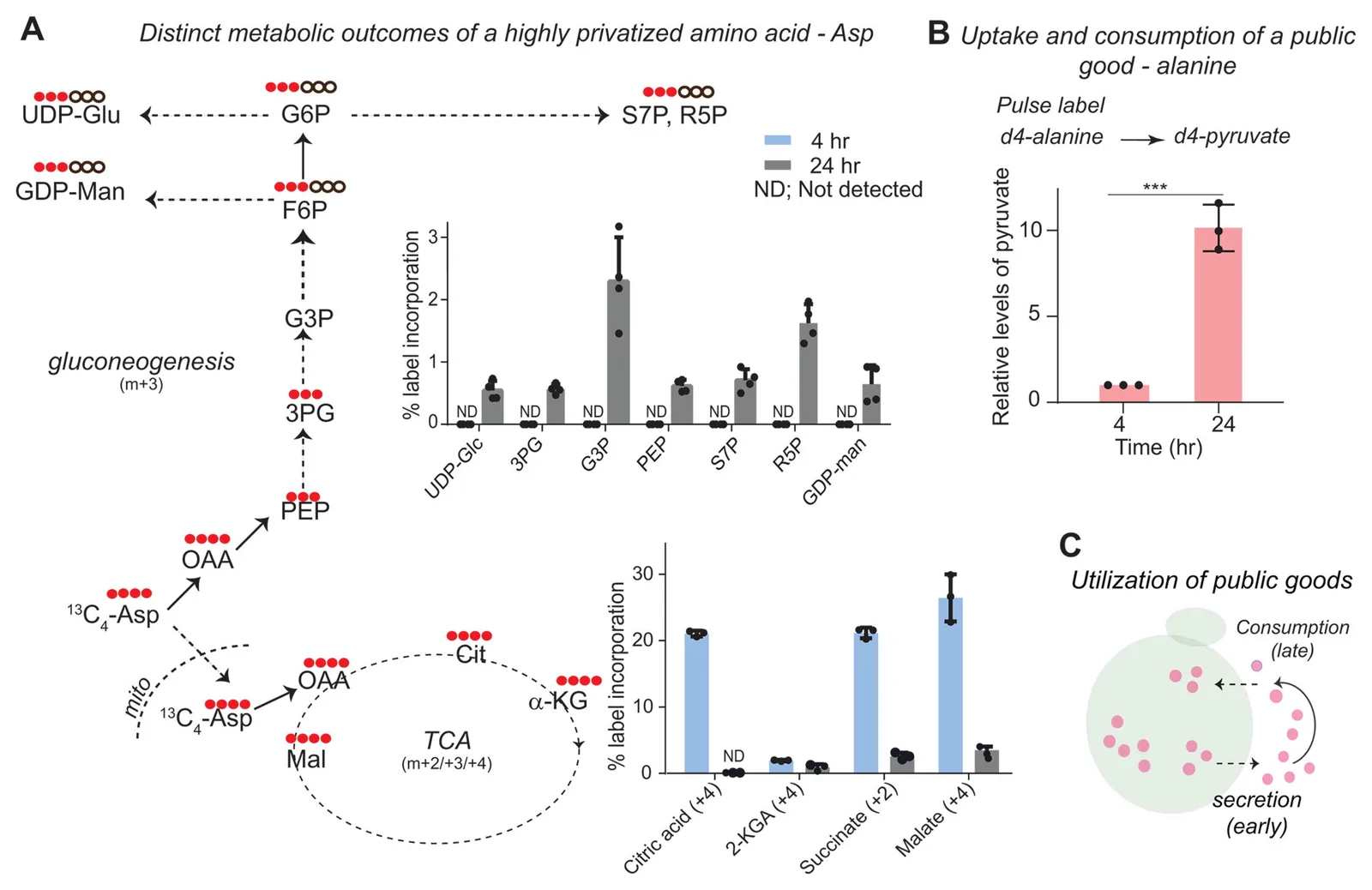
Functional allocation of a private and a public amino acid resource. (A) Demonstration of distinct allocations and continuous usage of a private good (Asp) at different times of growth. After 24 hours of growth, of the private amino acids, Asp shows the largest intracellular decrease, suggesting continuous usage. Experimental design to track the metabolic fates of aspartate, using a ^13^C_4_-aspartate pulse-label to monitor incorporation into distinct branches of carbon metabolism—the TCA cycle or gluconeogenesis. Aspartate converts to oxaloacetate, and the carbon from aspartate subsequently can be tracked as it incorporates into the TCA cycle or gluconeogenic intermediates by targeted LC-MS/MS. The relative utilization of aspartate as a carbon donor for intermediary metabolites in the TCA cycle, or for gluconeogenesis at 4 hours or 24 hours of growth is shown. At 4 hours, cells use aspartate to support the TCA intermediates, and at 24 hours mainly use aspartate for gluconeogenesis. Data are from three biologically independent experiments (*n* = 3), shown as mean ± SD. Also see Fig. S1. (B) Use of a public amino acid—Ala to support carbon metabolism during starvation. During glucose starvation, alanine can be converted to pyruvate (and glutamate). The experimental design (top): cells at 4 hours and 24 hours of growth were spiked with d_4_-alanine, and the relative d_4_-label conversion into pyruvate was estimated using targeted LC-MS/MS. The bar plot shows relative levels of d_4_-pyruvate. The pyruvate label coming from alanine considerably increases after 24 hours. Data are from three biologically independent experiments (*n* = 3), shown as mean ± SD, ***P < 0.001 (Student’s *t*-test). Also see Fig. S1. (C) The schematic illustrates a dynamic cycle of public good accumulation and consumption in yeast cells, particularly to sustain metabolism after starvation.

The private amino acids in yeast were a select subset—Arg, His, Lys, Asp, and Met. We further asked whether privatization was associated with the biosynthetic costs of the amino acid. Using established, comprehensive biosynthetic cost estimates for amino acids in this growth condition (24), we found that the privatized group (Arg, His, Lys, Asp, and Met) encompasses a broad range of costs (Fig. S1). While aspartate, arginine, and lysine have relatively low biosynthetic costs, methionine and histidine are energetically expensive (Fig. S1). This suggests that privatization in yeast is not determined by biosynthetic cost. In contrast, when evaluated based on cellular demand, the privatized amino acids consistently support high-flux pathways for protein and nucleotide synthesis, as well as distinct metabolic outputs (24) (Fig. S1). Additionally, while methionine levels are low in cells, the high demand for methionine comes primarily from s-adenosyl methionine, which is the primary biological methyl donor and an indicator of “metabolic charge” (38, 39). Aspartate in particular serves as a metabolically flexible precursor—it fuels the tricarboxylic acid (TCA) cycle, or enables gluconeogenesis via its conversion to oxaloacetate during glucose limitation, and supports nucleotide synthesis (17) (Fig. 4A and Fig. S1). The various uses of Arg, Lys, Met, and His (24, 32, 38) are also indicated in Fig. S6. Given that aspartate exhibited the most significant intracellular decline after 24 hours among the private amino acids (Fig. 3G), we experimentally explored its continuous utilization as a representative case of a privatized resource.

To estimate relative aspartate allocations during the course of growth, we performed stable isotope-based pulse-labeling experiments using ^13^C_4_-aspartate (Fig. 4A). We monitored label incorporation of ^13^C_4_-aspartate toward the TCA or gluconeogenesis at 4 hours (early) or 24 hours (late phase) of growth (Fig. 4A). At the 4-hour mark, the labeled ^13^C_4_-aspartate was predominantly incorporated into the intermediates of the TCA cycle (Fig. 4A). This indicates that aspartate is continuously converted to oxaloacetate to support TCA activity during the exponential growth phase. In contrast, at 24 hours, carbon from aspartate was incorporated substantially into gluconeogenesis intermediates (Fig. 4A), including UDP-Glc, PEP, G-3-P, GDP-Man, R-5-P, 3PG, and S-7-P. These data demonstrate how the continuous, adapting use of aspartate sustains diverse cellular needs, enabling metabolic transitions during growth.

As shown earlier, many public amino acids decreased in the extracellular environment after ~24 hours. Uptake and utilization have been observed in other microbes, including bacteria (23, 40). We next investigated the utilization of alanine, a representative public good that exhibited the largest extracellular depletion by 24 hours (Fig. 3G). In glucose-exhausted cultures, alanine can be catabolized to pyruvate, which can subsequently re-enter central carbon metabolism to sustain gluconeogenesis or the TCA cycle (41). We therefore assessed this uptake and traced the potential utilization of alanine in these cells. For this, we pulsed cells with stable isotope-labeled alanine (d4-alanine), and estimated (uptake and utilization) relative flux toward pyruvate formation at 4 hours and 24 hours. Pyruvate formation from alanine increased ~10-fold after 24 hours, compared to 4 hours (Fig. 4B). This indicates a shift in uptake and utilization of alanine toward pyruvate production later in growth phase (Fig. 4B). These findings demonstrate the functional cycle of a “consumed” public good: alanine is secreted substantially, only to be recovered later to support pyruvate-driven metabolism during late growth phases (Fig. 4B and C). This is summarized in the schematic in Fig. 4C. Collectively, these labeling data reveal two metabolic strategies: the continuous internal reallocation of private goods like aspartate to sustain essential flux, and the temporal use of public goods like alanine to adapt to a specific nutrient limitation.

### Nitrogen limitation drives a transition to amino acid privatization

**Fig 5:**
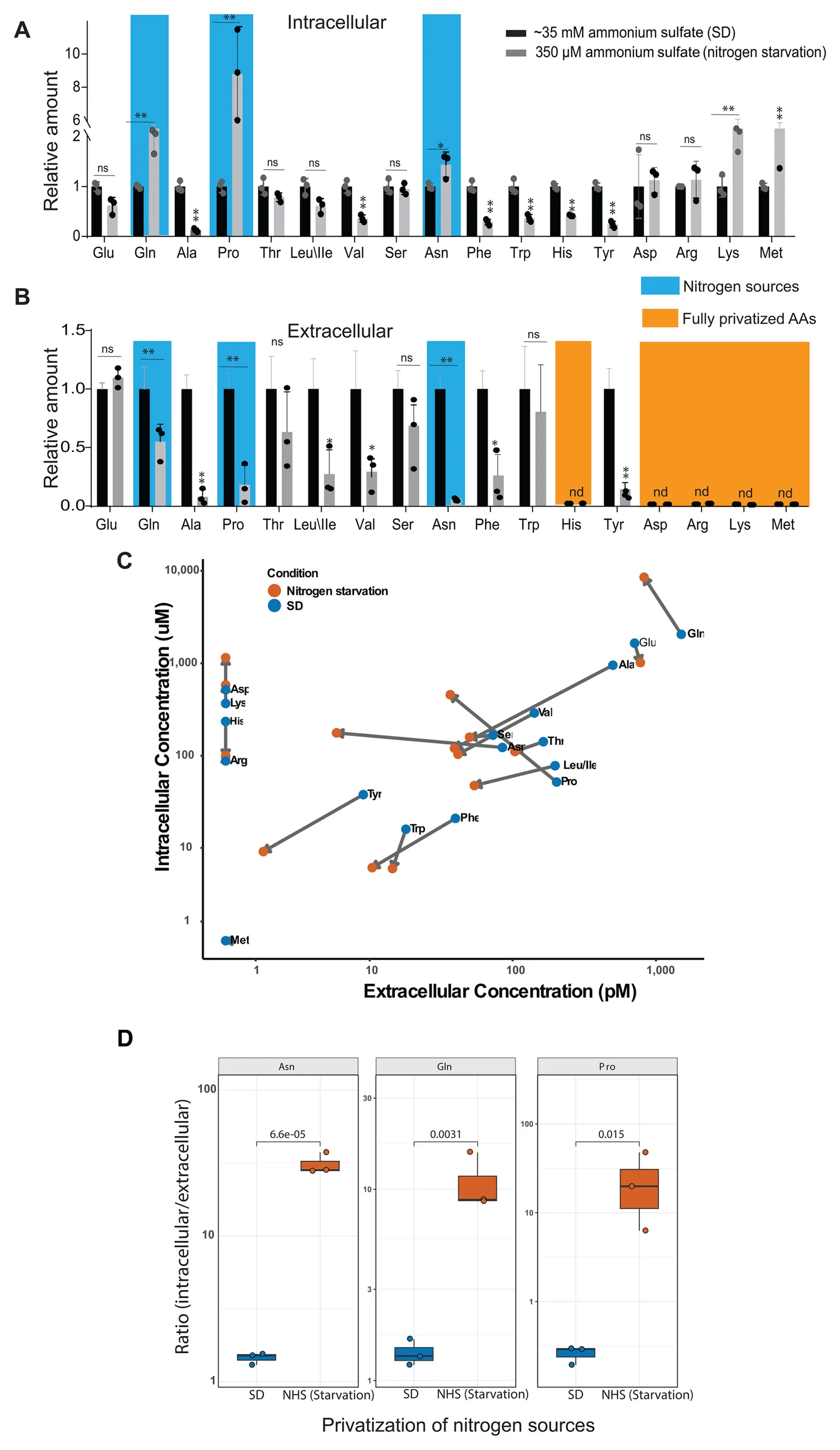
Nitrogen limitation drives a transition to amino acid privatization. (A) Bar plots showing the relative levels of intracellular amino acids from cells collected in standard minimal medium (SD, with ~ 35 mM ammonium sulfate), or from nitrogen starvation (ammonium sulfate limitation −350 μM), after 12 hours of growth. Intracellular amino acids were extracted and quantified as described earlier. Amino acids considered good (Gln and Asn) or poor (Pro) nitrogen sources for yeast are indicated with a blue box. *P < 0.05, **P < 0.01, ***P < 0.001 (Student’s *t*-test). Also see Fig. S2 for cell growth in these conditions. (B) The bar plots show relative amounts of extracellular amino acids from supernatants of cells grown in standard minimal medium, or nitrogen-limited medium after 12 hours of growth. Aminoacids considered good (Gln and Asn) or poor (Pro) nitrogen sources for yeast are indicated with a blue box. The privatized amino acids in standard minimal medium are shown with an orange box. *P < 0.05, **P < 0.01 (Student’s *t*-test). (C) A scatter plot, on a pseudo-log scale visualizing absolute intracellular amino acid amounts versus extracellular amino acid amounts. Cells grown in standard minimal medium are compared with those grown in nitrogen-starved medium. Most amino acids exhibit a clear and substantial decrease in extracellular amounts. (D) Increased privatization of nitrogen source amino acids. Intracellular-to-extracellular concentration ratios for Gln, Asn, and Pro in standard minimal medium (SD) versus nitrogen starvation (NHS). Data points represent independent biological replicates (*n* = 3). The significant increase in these ratios under starvation conditions reflects a cellular “privatization” strategy. Statistical significance was evaluated within each amino acid across conditions using independent two-sample *t*-tests, with exact *P*-values indicated on the graph.

Given this quantitative blueprint of absolute amino acid pools, we asked how nitrogen scarcity impacts the balance between production and secretion. Prototrophic yeast synthesize amino acids *de-novo* primarily through the assimilation of ammonium (42). To assess the impact of nitrogen availability on the distribution between amino acid production and secretion, we imposed a nitrogen limitation by reducing the ammonium sulfate concentration 100 fold, from 35 mM to 0.35 mM (a 100 fold reduction). Under this starvation condition, *S. cerevisiae* exhibited reduced but sustained growth kinetics (Fig. S2). At the 12-hour growth point, we observed a significant reorganization of the intracellular amino acid pools relative to standard media (Fig. 5A). Intracellular concentrations of glutamine, proline, asparagine, methionine, and lysine increased significantly; notably, glutamine and proline—already among the most abundant intracellular species—increased sixf-to eightfold (Fig. 5A). In contrast, valine, phenylalanine, tryptophan, histidine, and tyrosine concentrations decreased, while other amino acids remained largely invariant.

This accumulation pattern is significant given that glutamine and asparagine are preferred nitrogen sources, whereas proline is a poor nitrogen source typically utilized only under nitrogen-limited conditions (43), and lysine is not used by *S. cerevisiae* as a nitrogen source but is a high-nitrogen-containing amino acid. These data demonstrate that upon nitrogen limitation, the cell actively accumulates both high-quality and low-quality nitrogen-containing species. Furthermore, the total intracellular amino acid pool does not decrease commensurately with the 100-fold reduction of nitrogen in the medium, revealing a homeostatic mechanism for nitrogen retention.

Given this clear reorganization of intracellular amino acid pools under ammonium sulfate limitation (Fig. 5A), we next assessed what happens to extracellular amino acid pools. The extracellular amino acid profiles under ammonium limitation revealed a clear shift in partitioning (Fig. 5B). The extracellular concentrations of the typically public nitrogen sources—glutamine, asparagine, and proline—decreased substantially compared to standard media. Amino acids identified as private in standard media (arginine, aspartic acid, lysine, methionine, and histidine) remained strictly sequestered and were undetectable in the supernatant (Fig. 5B).

A comparative analysis of intracellular and extracellular amounts (Fig. 5C) shows that nearly all amino acids exhibit a decrease in extracellular availability despite maintaining or increasing their intracellular concentrations. Finally, the intracellular/extracellular ratio factors for glutamine, asparagine, and proline significantly increased during nitrogen starvation (Fig. 5D), revealing a clear privatization of key nitrogen sources/reserves. These results establish that nitrogen limitation triggers a transition from metabolite secretion to increased privatization and storage of nitrogen reserves, and maintaining intracellular amino acid amounts comparable to cells in standard media.

### Public good auxotrophs utilize amino acids from conditioned media to sustain robust growth

**Fig 6:**
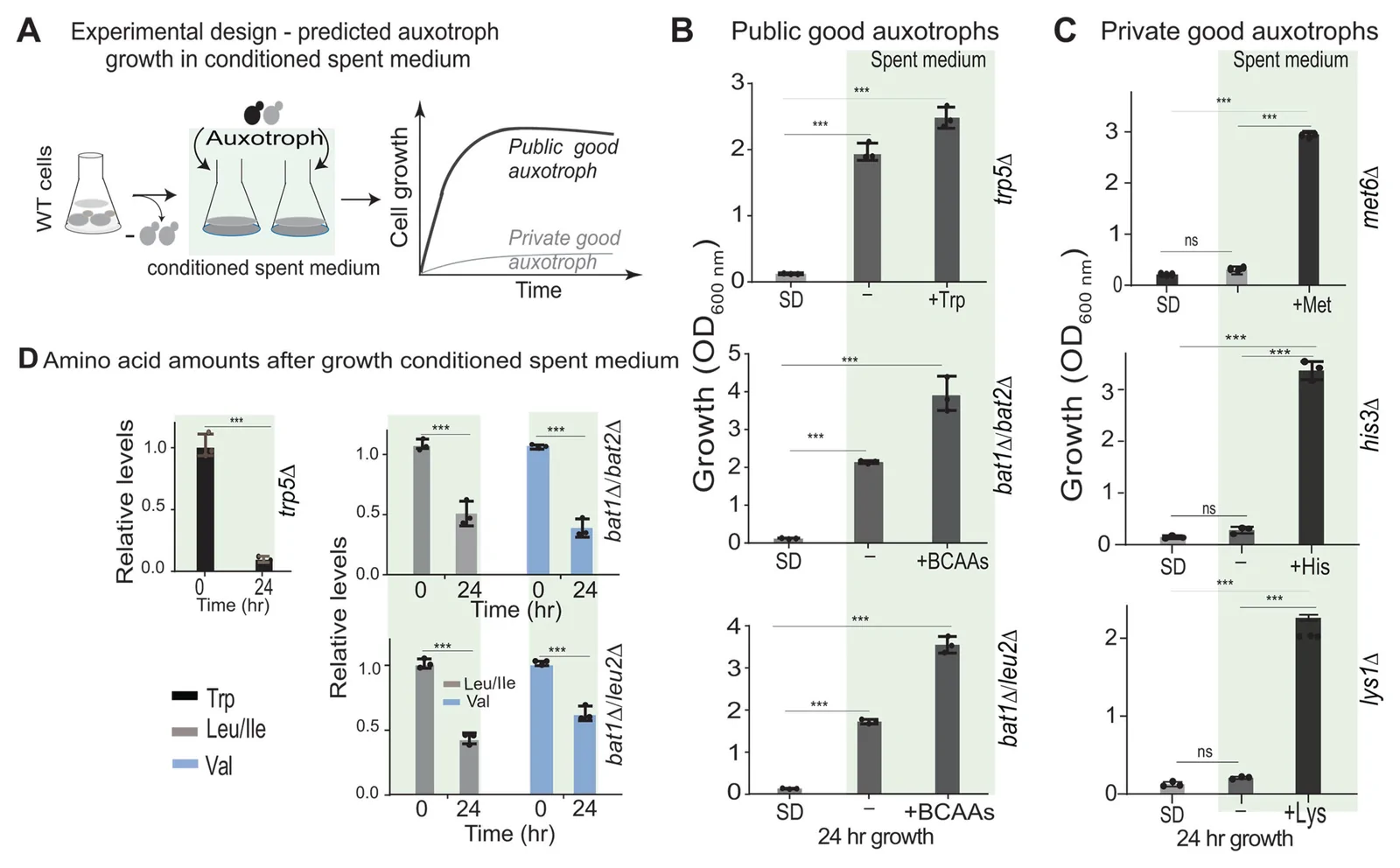
Public good auxotrophs utilize amino acids from conditioned media to sustain robust growth. (A) Experimental design to assess auxotrophic growth in spent medium. Spent medium after 24-hour growth of wild-type cells was collected and supplemented with glucose and ammonium salts to make conditioned supernatant. Subsequently, the specified public good auxotroph or private goods auxotroph is allowed to grow in conditioned supernatant. The predicted growth of a public good amino acid auxotrophic cell versus a private public good amino acid auxotrophic cell in conditioned supernatant is also illustrated. Also see Fig. S3 for auxotrophic cell growth. (B) Growth of public good auxotrophs in spent medium. Auxotrophs of the following public good amino acids*—trp*5Δ (auxotrophic for tryptophan), Δ*bat1*Δ*/*Δ*bat2*Δ (auxotrophic for all branched-chain amino acids), *bat*1Δ/*leu*2Δ (alternate of auxotrophic for BCAAs)—were grown in conditioned supernatant. As controls, these cells were also grown in conditioned supernatant that was supplemented with 2 mM of the amino acid relevant to the specific auxotrophy. (C) Growth of private good auxotrophs in conditioned supernatant. Auxotrophs of the following public good amino acids*—met6*Δ (auxotrophic for methionine), *his3*Δ (auxotrophic for histidine), *lys1*Δ (auxotrophic for lysine)—were grown in spent medium supplemented with glucose and ammonium salts only. As controls, these cells were also grown in conditioned medium that was supplemented with 2 mM of the amino acid relevant to the specific auxotrophy. (D) Relative amounts of the specific extracellular amino acid (relevant to the auxotrophy) after 24 hours of growth in conditioned supernatant. Auxotrophic cells were grown in conditioned supernatant (as in panel B). The bar plots show the relative amounts of the indicated amino acid in the extracellular environment after the specified amino acid auxotroph was grown in conditioned supernatant. The auxotrophic strains used were: Δ*trp*5, Δ*bat1*Δ/*bat2*Δ, and *leu*2Δ/*bat1*Δ. Each auxotroph was grown in spent medium of wild-type cells, and the amount of the specific amino acid was measured at 0 hour and after 24 hours. Data in all panels are from three independent biological replicates, represented as means ± SD, *n* = 3. ***P < 0.001 (Student’s *t*-test).

The quantitative determination of intracellular and extracellular amino acid pools leads to the hypothesis that “public” amino acids are available for utilization by cells in the population. If so, auxotrophic strains deficient in the biosynthesis of public goods should show robust growth by exclusively utilizing the extracellular supply provided by prototrophic cells. Conversely, these data would predict that auxotrophs of private good amino acids would not be able sustain growth in spent medium (Fig. 6A).

To test this, we designed experiments to investigate whether the secreted amino acids in spent media could sustain the growth of auxotrophic strains of public or private good amino acids (Fig. 6A). We generated specific auxotrophic strains (Fig. S3). Public good auxotrophs included *bat1*Δ*/bat2*Δ and *leu2*Δ*/bat1*Δ (branched-chain amino acids), and *trp5Δ* (tryptophan) (Fig. S3). Private good auxotrophs included *met6*Δ (methionine), *lys1*Δ (lysine), and *his3*Δ (histidine) (Fig. S3). Our experimental design was as follows—we first prepared “conditioned spent medium,” by growing wild-type cells in synthetic minimal medium for 24 hours, collecting the culture supernatant, and supplementing it with glucose and ammonium sulfate to ensure carbon and nitrogen sufficiency (Fig. 6A). We then assessed the growth of the different auxotrophic strains in this conditioned spent medium (Fig. 6A). The conditioned spent medium is a complex environment containing depleted nutrients and accumulated metabolites. For this experiment, by using this exact same medium for both our experimental and control groups, we ensured consistency, with the only variable being the addition of the specific amino acid, while maintaining a complex environment. For the public good auxotrophic strains, we assessed the growth of *bat1*Δ/*bat2*Δ, *leu2*Δ/*bat1*Δ, and *trp5*Δ cells in this conditioned spent medium, and this growth in conditioned medium was compared to positive controls supplemented with 2 mM of the required amino acids directly in the conditioned spent medium (Fig. 6B). Note: as controls, we include data showing that the growth of these auxotrophs in standard minimal medium, supplemented only with the respective amino acid, is indistinguishable from prototrophic cells (Fig. S4). Notably, the conditioned spent medium supported the growth of these public good auxotrophic strains, at times as effectively as the supplemented controls (Fig. 6B). These findings reveal that the public good amino acids are produced/secreted in substantial excess to support the effective growth of their respective auxotrophic cells (Fig. 6B).

We next assessed the growth of private good auxotrophs (*met6*Δ, *his3*Δ, and *lys1*Δ) in conditioned supernatant (Fig. 4C). In contrast to the public good amino acid auxotrophs, the private good amino acid auxotrophic strains showed negligible growth in conditioned supernatant, in the absence of exogenous supplementation of the respective amino acids (Fig. 6C). This confirms that methionine, lysine, and histidine are sufficiently sequestered within cells, and are not available externally to sustain their growth (Fig. 6C).

Finally, we quantified the depletion and utilization of these public amino acids from conditioned spent medium by their respective auxotrophs. For this, we assessed the relative levels of specific amino acids (tryptophan, leucine/isoleucine, and valine) in conditioned spent medium after 24 hours of growth of the indicated auxotrophic strain (Fig. 6D). After 24 hours of growth, tryptophan, leucine/isoleucine, and valine levels were significantly reduced (Fig. 6D), confirming uptake and utilization. Collectively, these findings validate the classification of amino acids into distinct economic categories and demonstrate that public goods can effectively support the growth of dependent individuals in a community.

### Impact of auxotrophy on intracellular and extracellular amino acid pools

With this quantitative understanding of intracellular and extracellular amino acid amounts, we investigated how the exogenous supply of a required amino acid impacts the global intracellular and extracellular amino acid pools of an auxotroph. Specifically, we asked whether supplying a single amino acid alters the production of other public or private goods. We tested this for one representative each for public and private good auxotrophs (*trp5*Δ—public good auxotroph, and *lys1*Δ —private good auxotroph). We selected these two examples because single-gene deletions (of *TRP*5 and *LYS1*, the first biosynthetic steps of the respective pathways) result in robust, clean auxotrophs that have no growth without amino acid supplementation and fully normal growth when the amino acid is supplemented. When supplemented with 2 mM of the respective amino acid, both auxotrophs show growth indistinguishable from wild-type, prototrophic cells (Fig. S4).

We compared the intracellular and extracellular amino acids present in these cells/media to those of wild-type cells (Fig. S5 and S6, respectively). In the private good auxotroph (*lys1*Δ), the intracellular amino acid levels for most amino acids show only a small but significant decrease (Fig. S5), while glutamate, threonine, and proline are maintained similar to the prototrophic strain (Fig. S5). In contrast, the extracellular amino acid pools showed a distinct trend. Most amino acids did not significantly change compared to prototrophic cells. However, these cells interestingly showed increased secretion/amounts of glutamine (Fig. S6). Lysine is a high-demand amino acid directly derived from glutamate (24), and as a nitrogen-rich amino acid, is deeply integrated with nitrogen metabolism. A similar correlation (of increased glutamine when lysine amounts increase) has been observed in *Corynebacterium glutamicum* (44). Additionally, in yeast, lysine supplementation feedback inhibits the early steps in lysine biosynthesis (45), to increase pools of the precursor 2-oxoglutarate, and also spares nitrogen otherwise used for lysine biosynthesis, to likely contribute to the observed increase in intracellular pools of glutamine. These data suggest that by reducing the need to make this specific, high-demand amino acid, cells will increase their allocation toward producing glutamine, to make it available in large amounts externally.

We carried out similar experiments with a public good auxotroph (*trp5*Δ), grown with tryptophan supplemented, and compared intracellular and extracellular amino acid levels. Contrastingly, in this case, neither intracellular nor extracellular amino acid amounts changed significantly from the prototrophic cells (Fig. S5 and S6, respectively). This lack of a metabolic shift suggests that prototrophic cells already synthesize an excess of this public good, and therefore restoring its supply exogenously has a minimal effect on the production-consumption cycles for other amino acids. Collectively, these results reiterate that the metabolic impact of auxotrophy is dependent on the specific demand for that amino acid.

### Co-cultures of only public-public auxotroph pairs show robust growth

**Fig 7:**
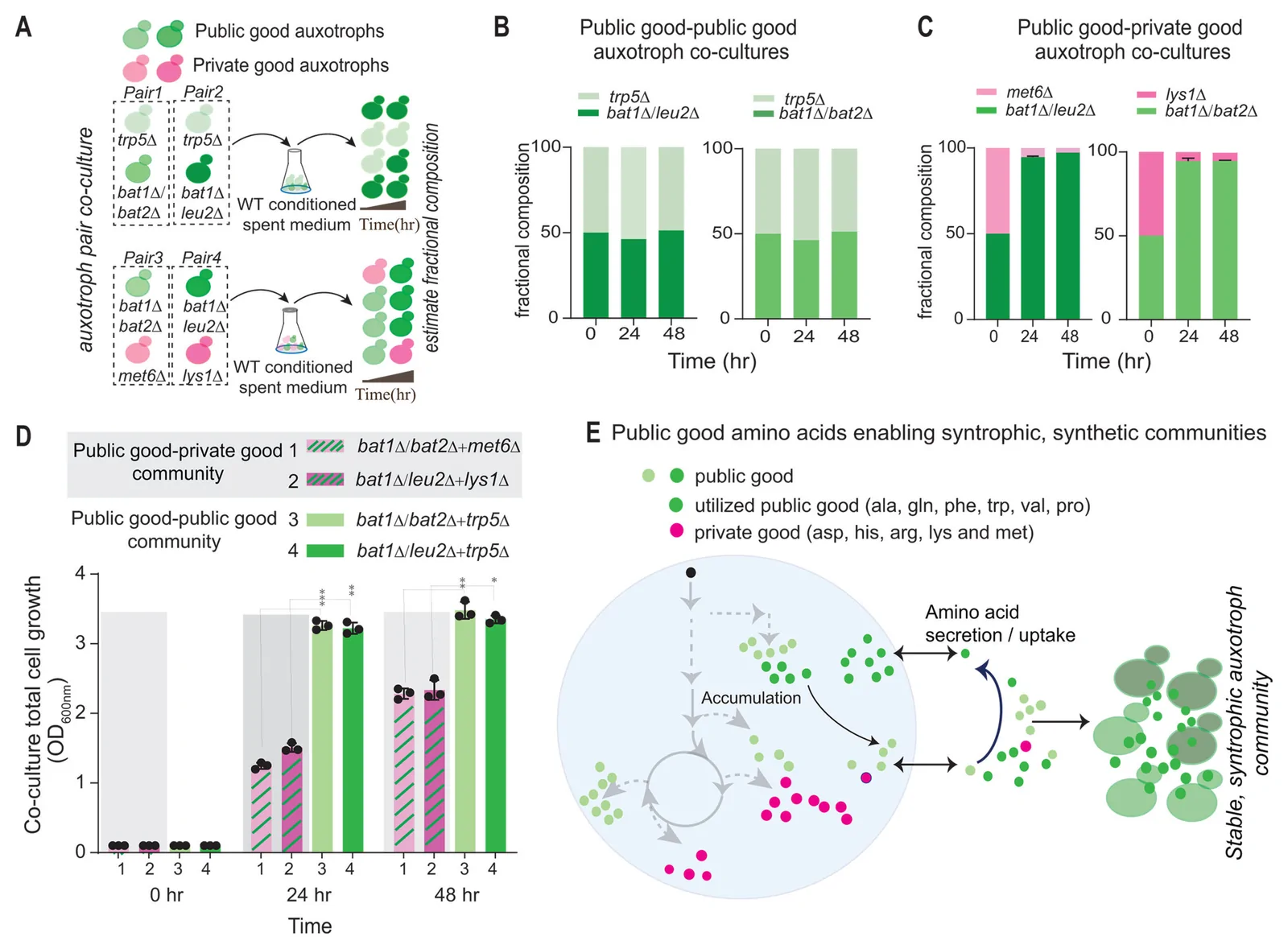
Co-cultures of public-public good auxotroph pairs show robust growth. (A) Experimental design to assess the growth of auxotroph pairs of co-cultures seeded in conditioned medium. The indicated combinations of public good-public good auxotroph pairs or public good-private good auxotroph pairs were co-cultured in conditioned medium (spent medium supplemented only with glucose and ammonium salts). The fractional (proportional) composition of each strain in the paired culture, as well as the total biomass attained, after the indicated time of growth, is then estimated. (B) Public good-public good auxotroph pairs maintain proportional growth: Bar plots displaying the fractional composition of each strain in the indicated paired co-cultures of public good-public good auxotrophs. The paired auxotrophic cell co-cultures tested were *trp5*Δ *+ bat1*Δ*/leu2*Δ and *trp5*Δ *+ bat1*Δ*/bat2*Δ. (C) Private good auxotrophs are outcompeted by public good auxotrophs: Bar plots displaying the fractional composition of each genotype in paired co-cultures of public good-private good auxotrophs. The paired auxotrophic cell co-cultures were *met6*Δ *+ bat1*Δ*/leu2*Δ, and *lys1Δ + bat1*Δ*/bat2*Δ. (D) Public good-public good auxotrophic pairs show synergistic growth. Total biomass at the indicated time after growth of public good-public good auxotrophic pairs, compared to public good-private good auxotrophic pairs. Each paired community was grown in the same conditioned supernatant. The public good-private good auxotrophic pair communities tested were *bat1*Δ*/bat2*Δ *+ met6*Δ and *bat1*Δ*/leu2*Δ *+ lys1Δ*. The public good-public good auxotrophic pair communities tested were *bat1*Δ*/bat2*Δ *+ trp5*Δ and *bat1*Δ*/leu2*Δ *+ trp5*Δ. Additionally, the individual growth of each auxotroph in conditioned supernatant is shown earlier in Fig. 6. (E) A model illustrating a dynamic private and public good amino acid economy in yeast and how it can shape exchange-based communities. Amino acid production, secretion, accumulation, and exchange are illustrated, and distinct amino acids are private (pink balls) or public (green balls) goods. This definition changes temporally, although private amino acids tend to remain privatized. Auxotrophic strains of public good amino acids are effective in forming exchange-based communities, given the surplus presence of public amino acids. Data in panels A–D are from three independent biological replicates (*n* = 3), represented as mean ± SD. *P < 0.05, **P < 0.01 ***P < 0.001 (Student’s *t*-test).

Similar to other microbes, yeast can form exchange-based auxotrophic communities (6, 11, 46); yet, underlying metabolic rules driving these interactions remain unresolved. Our quantitative data now suggest that auxotrophs of surplus public goods might establish effective communities based on the mutual exchange of surplus metabolites. We previously demonstrated that public good auxotrophs exhibit robust growth by using the corresponding amino acid present in the extracellular medium. We therefore tested whether effective pairs of co-cultures could be established via complementary amino acid exchange, using paired co-cultures of auxotrophs of two public amino acids, or public-private amino acid (Fig. 7A). We inoculated each paired combination with 50:50 ratios of each strain in low cell density in conditioned supernatant, and monitored co-growth in this medium.

The auxotrophic-pairs used were as follows: public-public auxotroph pairs (*bat1*Δ*/bat2*Δ *+ trp5*Δ and *bat1*Δ*/leu2*Δ *+ trp5*Δ) and public-private auxotroph pairs (*bat1*Δ/*bat2*Δ *+ met6*Δ and *bat1*Δ*/leu2*Δ *+ lys1*Δ) (Fig. 7A). Do note that as monoculture controls for each auxotroph, the growth of individual auxotrophs in the wild-type conditioned medium was included earlier (Fig. 6B for public good auxotrophs, and Fig. 6C for private good auxotrophs), and establish that public good auxotrophs utilize the baseline amino acids in the conditioned medium to sustain initial growth, whereas private good auxotrophs cannot, as those specific amino acids are sequestered within the prototrophic cells.

To accurately estimate the fraction of each auxotroph, each strain was engineered with a constitutive, fluorescent protein-label (mCherry or mNeonGreen), in the indicated combinations (Fig. 7B and C). We then monitored the fractional composition (of each auxotroph) over time (Fig. 7B and C). In all public good-public good auxotrophic pairs of co-cultures, we observed robust growth and a stable population of each strain (Fig. 7B). For each of these pairs tested, a constant ratio of each genotype was maintained over 48 hours (Fig. 7B), indicating a stable, mutual-exchange-based community. In contrast, for public good-private good auxotrophic co-culture pairs, the fractional composition of each strain rapidly changed (Fig. 7C). The public good auxotrophic strains utilized the conditioned supernatant and grew, increasing in fractional composition, while private auxotrophic strains failed to thrive in the co-culture (Fig. 7C), with their relative population abundance decreasing to <5% (Fig. 7C).

We subsequently investigated whether pairing public good-public good auxotrophs in co-cultures led to increased cell numbers and total biomass via amino acid exchange, relative to a public-private good auxotroph pair (Fig. 7D). To determine whether this growth was sustained merely by the initial nutrient content of the conditioned supernatant or necessitated active continuous secretion, we monitored total cell numbers across the co-culture combinations over time (Fig. 7D). We observed that the public good-public good auxotrophic pairs exhibited robust growth, reaching significantly higher total biomass in 24 and 48 hours of co-culture (Fig. 7D). These data indicate that pairing public good-public good auxotrophs together leads to a significant increase in total cell number and biomass, unlike with public-private co-cultures that rely exclusively on available amino acids in conditioned supernatant (Fig. 7E). The public good-public good auxotroph co-cultures consistently outperform public-private co-cultures in biomass formation, through a robust exchange of public goods (Fig. 7E).

Collectively, these data underscore the importance of identifying public good amino acids, through which pairs of auxotrophic cells can be co-cultured, leading to increased biomass (Fig. 7E). The use of public goods driven by production in excess of individual demand could therefore enhance collective cell population growth (Fig. 7E).

## Discussion

In this study, we provide a comprehensive, quantitative analysis of the temporal dynamics governing amino acid synthesis, secretion, and consumption throughout a 24-hour yeast growth cycle, revealing a dynamic intracellular and extracellular amino acid environment (Fig. 7E). We demonstrate that peak amino acid biosynthetic flux is restricted to the early stages of batch culture growth and show that transcriptional programs induced at later time points represent a starvation-induced response—an attempt to restore biosynthetic flux that is not metabolically supported (Fig. 1). Subsequently, we built a quantitative blueprint of absolute intracellular and extracellular amino acid amounts and temporal dynamics, identifying that the relative proportions of amino acids change substantially over different phases of growth (Fig. 2). Two clear observations emerge: First, the intracellular pools differ by ~3–4 orders of magnitude between the most and least abundant, and second, that the intracellular stoichiometric proportions of amino acids are dynamic. Notably, the extracellular environment was entirely distinct from the intracellular pools (Fig. 2) in both absolute magnitude and composition. Some amino acids were minimally present in the external environment and remained sequestered within cells. Collectively, we have established a highly quantitative, rigorous blueprint of the intracellular and extracellular amino acid economy of yeast. This quantitative foundation can critically inform any future basic or applied studies built on microbial amino acid exchange.

Our analyses can now identify and demarcate amino acids that tend to be privatized by cells, versus those that are made publicly available and therefore more easily exchanged or utilized (Fig. 3). The private amino acids support diverse metabolic processes and sustain growth. In contrast, public goods can be grouped into two functional categories: those secreted in surplus and those initially produced in excess but subsequently re-imported and consumed as nutrients deplete. Furthermore, we established that the degree of privatization is a dynamic response to nutrient availability. Under ammonium sulfate limitation, yeast start to sequester and privatize other amino acids—particularly glutamine, proline, and asparagine, which are high-value nitrogen sources (Fig. 5). This transition from public to private status reveals that yeast strategically shift resource management during nitrogen scarcity. These findings complement recent studies that show that yeast cells hold excess metabolic reserves that collectively change upon nitrogen limitation (26).

The extent to which surplus public good amino acids might support the formation of effective, complementary auxotrophic pairs of cells that are truly exchange-based communities remains challenging to fully establish. This challenge has been compounded by a lack of any quantitative information regarding the amounts of amino acids made versus secreted out of the cell, and how much of that can be utilized. From the presented data, we find that auxotrophs of public goods can grow effectively using resources exclusively obtained from their extracellular environment, and that public good auxotrophs are also ideally suited to form stable, exchange-based synthetic communities (Fig. 7). It is of course important to note that this study is limited to an *in vitro* demonstration of how two auxotrophs, for easily available public amino acids, can be paired to lead to increased total biomass while maintaining stable individual populations. The conditioned medium used in the experiments acts as a catalyst; once growth is initiated, the public-public auxotrophs can grow effectively in this fairly short time window to exceed the carrying capacity of the initial medium for any one individual strain. Conversely, the public-private co-cultures fail to establish this loop (due to the lack of secreted private goods) and their growth is limited by only what the initial medium can support, and a collapse of the private-good auxotroph population (Fig. 7C). Additionally, it is important to acknowledge that in these collective growth experiments, with the private amino acid auxotrophs (that do not grow), we cannot distinguish if there is a complete absence or insufficient concentration of the respective amino acid. We presume that it is an insufficient concentration. Trace amounts may transiently support maintenance, but are insufficient to meet the demand required to sustain growth (or support extensive cross-feeding). Consequently, the cells are unable to harvest enough of these limited private goods from the environment, leading to ineffective metabolic complementation and the collapse of the population. Collectively, this quantitative data set allows us to establish hierarchies in a cellular amino acid economy, built on biosynthesis, secretion, and consumption over short time-scales, as illustrated in the schematic Fig. 7E. This also highlights a dynamic intracellular and extracellular amino acid economy that has been underappreciated (Fig. 7E).

These findings advance our quantitative understanding of the cellular amino acid economy. Despite widespread use of laboratory models like yeast or *E. coli*, our quantitative understanding of the intracellular and extracellular amino acid economy remains unexplored. Recent work suggests that in minimal medium, yeast cells secrete amino acids at the exponential phase, which increases in the early stationary phase (47). Previous studies have noted that intracellular amino acids were synthesized on demand during the yeast cell cycle, or during yeast metabolic cycles (13, 27, 31). Our data provide complementary, quantitative insights into the identity and magnitude of synthesized amino acid pools. Additionally, while existing studies have been limited to static snapshots of certain intracellular or extracellular metabolites (25, 48, 49), our temporal, quantitative analyses of production, accumulation, consumption, and exchange substantially expand the scope of these studies. Given that cells have dramatically different amounts of individual amino acids, or amino acid reserves (25, 26), and that the amino acid economy functions as a demand-based economy (24), the metabolite exchange observed must therefore function within those constraints (47). By providing a quantitative blueprint of amino acid production and absolute concentrations present in the intracellular and extracellular environment, this work not only complements these past studies, but will permit the informed design of effective, amino acid exchange-based communities. We acknowledge that this study does not consider the contributions of amino acid salvage and recycling to the amino acid economy. This is technically very challenging to perform. However, using this base of amino acid production, accumulation, and uptake in this study, future investigations can further distinguish relative contributions of *de-novo* and salvage pathways to these sources. Additionally, an independent direction of inquiry is the role played by different amino acid transporters in maintaining the balance of secretion vs uptake, which is largely unknown. Finally, the results of this study reveal aspects of the amino acid economy of prototrophic yeast growing in glucose and a preferred nitrogen source. Many aspects of these findings will apply to prototrophic microbes growing with a preference for similar carbon/nitrogen sources. Therefore, our data provide a valuable foundation for inquiry in model microbial factories, using defined conditions in laboratory settings. Eventually, the dynamics of the cellular amino acid economy is a feature of the inherent constraints imposed by growth, as well as the metabolic flexibility of that cell. In light of the reductionist findings in this study, it remains to be determined what range and limits are for amino acid amounts inside different types of cells, and how changes in one amino acid result in the induction or reduction of another amino acid.

Cooperative interactions based on syntrophic metabolic exchange and cross-feeding enable groups of cells to build metabolic efficiencies, drive auxotrophic cell populations toward new metabolic imbalances and interactions, or even improve lifespan and survival (3, 9, 10, 47, 50). Consequently, designing and engineering synthetic communities based on syntrophic exchange has become an area of considerable interest, for the obvious potential it holds for biotechnological applications. Yet, many efforts to design exchange-based microbial consortia for biotransformations fall short of meeting expectations (6, 12, 23, 51–53), in part due to challenges in quantitatively untangling metabolic interactions. Through this study, by quantifying intracellular and extracellular amounts and identifying which (public good) amino acids are effectively exchanged, we provide a first step toward rationally designing effective synthetic, syntrophic co-cultures. Complementary public-public co-cultures outperformed public-private co-cultures with respect to growth. This quantitative, bottom-up approach on inherent biochemical constraints for producing and secreting excess amino acids can be a complementary approach to adopt while building synthetic microbial consortia for potential bioproduction applications. Such quantitative information becomes critical for identifying supply versus demand constraints for distinct amino acids (24), and defines amino acids that are effectively exchanged to such synthetic communities. Context-specific, quantitative insights into their production, secretion, and consumption cycles can identify resource bottlenecks and suggest engineering strategies to overcome these bottlenecks. This quantitative approach toward defining the amino acid economy broadens our understanding of cellular resource allocation strategies and provides a rational design of metabolically engineered consortia as cell factories.

## Materials and Methods

### Yeast growth and media conditions

A prototrophic, haploid (CEN.PK mat a) strain of *Saccharomyces cerevisiae* 36 was used in all the experiments and was maintained in YPD medium containing yeast extract (1%), peptone (2%), glucose (2%), and agar (2%). For growth experiments, a primary culture of *S. cerevisiae* was grown overnight in YPD nutrient broth in a screw-capped tube, and the primary culture was washed and diluted to an OD_600_ ~0.10 of cells in the growth medium. The synthetic defined medium (SD) used contained nitrogen base without amino acids and 2% glucose as the sole carbon source and ~ 35 mM ammonium sulfate as the sole nitrogen source. Cells were incubated in an orbital shaker operating at 240 rpm at 30°C. The list of strains and plasmids used in this study are provided in Table S1 and S2.

### Intracellular amino acid extraction and detection

Intracellular amino acids were extracted and quantitatively estimated using targeted LC-MS/MS approaches described earlier (35), with samples collected from cultures grown in synthetic defined minimal medium over a 24-hour growth period. Specifically, at time points of 2, 4, 8, 12, and 24 hours, equal numbers of cells (~2 × 10^7^) were quenched in extraction buffer (60% methanol), extracted in 75% ethanol, and dried down using a speed vacuum (rotary evaporator). Metabolites were dissolved in 300 µL mass spectrometry grade water, and 10 µL sample was injected for LC-MS/MS and separated using Synergi 4-µm Fusion-RP 80 Å (150 × 4.6 mm) LC column (Phenomenex, 00F-4424-E0). Solvents used for amino acid and TCA derivatives are 0.1% formic acid in water (solvent A) and 0.1% formic acid in methanol (solvent B). An AB Sciex QTRAP 5500 with Shimadzu Nexera series UPLC system was used in all studies. Detection of amino acids was done in positive polarity mode. Mass spectrometry data were acquired using Analyst 1.6.2 software (Sciex). For analysis, MultiQuant version 3.0.1 and Peak View version 2.0 were used. A table with Q1 and Q3 parameters for all metabolites, including labeled and unlabeled forms, is provided (Table S3). Pure standards of amino acids were used for absolute quantification, after establishing linear calibration curves over six orders of magnitude (worksheet File S1). To calculate intracellular amino acids per cell, the following information was considered: 1.0 mL of 1.0 OD_600_ culture = ~2 × 10^7^ cells, the volume of a single yeast cell = ~40 × 10^−15^ L. The absolute intracellular concentration of the respective amino acid (µM) = 1 × (cell concentrations in µM/L)/(molecular weight of individual amino acids/AA) × 2 × (10^7^ × 40 × 10^−15^ L).

### Extracellular amino acid extraction and quantification

*S. cerevisiae* cultures were grown in a minimal medium from early to the post-diauxic growth phase, as described earlier. All experiments were carried out using 30 mL of medium in shake flasks. At specific time points (2, 4, 8, 12, and 24 hours), 1 mL culture was collected. The culture was centrifuged for 5 minutes at 7,000 rpm at RT, and 250/500 µL supernatant was used for extracellular metabolite extraction. The extracellular amino acids were extracted with 75% ethanol. The extracted metabolites were then vortexed for 1.0 minutes and placed on ice for a few minutes. The samples were centrifuged at 16,000 rpm for 12 min at RT, and 750 µL of the supernatant was collected in new Eppendorf tubes, which were dried using a speed vacuum. The dried metabolites were stored at –80°C until mass spectrometry analysis was performed as described above and earlier (35). All peak intensity values from the experiments are provided in the supplementalworksheet. The final molar concentrations were calculated for each sample, using the final extracellular medium volume as 30 mL.

### Auxotrophic strain generation

In the prototrophic background, we introduced multiple knockouts through homologous recombination of specific genes using standard drug selection cassettes (54). Correct clones were verified using PCR with specific primers. The list of auxotrophic strains used in this study is provided in Table S2.

### ^13^C_4_-aspartate utilization flux measurement by LC-MS/MS

The ^13^C_4_-aspartate utilization flux was measured by tandem mass spectrometry to investigate the incorporation of carbon from aspartate into intermediates of the TCA cycle, gluconeogenesis, or other metabolites. Cells were grown in a minimal medium (SD; synthetic defined medium) for 4 and 24 hours, pulsed with 1 mM ^13^C_4_-aspartate (Cambridge Isotope Laboratories, CLM-1801-0.25G), and incubated for 20 minutes. Intracellular metabolites were extracted from ~ 1.0 OD cells, derivatized, and analyzed as described earlier (35). Detection of label incorporation into TCA intermediates was done in positive polarity mode. Parent (Q1) and product ions (Q3) that were used for detection of labeled metabolites are provided in Table S3. The total ^13^C_4_-aspartate label incorporation was calculated as the sum of all individual peak areas coming from ^13^C_4_-aspartate incorporated and detected for each specific metabolite. Relative label incorporation was calculated by normalizing with respective control values in each set.

### Measurement of ^15^N label incorporation flux from (^15^NH_4_)_2_SO_4_ into amino acid by LC-MS/MS

^15^N ammonium sulfate label incorporation into newly synthesized amino acids was measured using tandem LC/MS/MS, using methods described earlier (35). The culture was initially grown in minimal medium at 2, 4, 8, 12, and 24 hours before being pulsed with 50% ammonium sulfate (^15^NH_4_)_2_SO_4_; CIL, Sigma-Aldrich 299286-20G| and incubated for 20 minutes. This means that during the ammonium sulfate flux experiment, 18 mM of ^15^N-labeled (and 18 mM unlabeled) ammonium sulfate was added to the cultures. For the 24-hour time point, 50% unlabeled ammonium sulfate was added for 30 minutes, and then 50% labeled ammonium sulfate was added and incubated for 20 minutes. Subsequently, metabolites were extracted as described earlier, and ^15^N-labeled amino acids (coming from ^15^N-labeled ammonium sulfate) were estimated, as described. Detection of 15N-label incorporation into the specific amino acid was carried out using the Q1 and Q3 masses described in Table S3. Statistical significance was calculated using an unpaired Student’s *t*-test as indicated.

### Measurement of deuterated alanine (d4-alanine) conversion to pyruvate by LC-MS/MS

Deuterated alanine (d4-alanine) uptake and incorporation into pyruvate were measured using tandem mass spectrometry. The culture was initially grown in minimal medium at 4 and 24 hours before being pulsed with 1 mM d4-alanine (Cambridge Isotope Laboratories, 299286-20G) and incubated for 5 minutes. Subsequently, metabolites were extracted as described, and d4-labeled pyruvate (coming from d4-alanine) was estimated, similar to as described above. Detection of d4-pyruvate used the Q1 and Q3 masses described in Table S3. Statistical significance was calculated using an unpaired Student’s *t*-test.

### Luciferase reporters for Gcn4 activity

To measure Gcn4 activity, we used a well-established Gcn4-luciferase reporter system (Table S1) as described earlier (24, 32). Cells expressing the reporter were grown in the presence of antibiotic (G418), and the relative luciferase expression was estimated in cells grown in the respective minimal medium and collected at the indicated time. For the experiment, overnight cultures with cells expressing the reporter were collected and washed twice in minimal medium and used to set up a fresh culture in a 250 mL flask, with 100 mL synthetic defined medium with 2% glucose. One group of flasks served as a control with 2 mM amino acid (+AA) supplemented. In the control groups, after 2 hours, 2 mM amino acid was added, and after 4 hours, cells were collected at an OD_600_ ~10. Biological replicates of samples at 2, 4, 8, 12, and 24 hours were collected. Collected cell pellets were washed with 300 μL lysis buffer containing 1× PBS (137 mM NaCl, 2.7 mM KCl, 10 mM Na_2_HPO_4_, 1.8 mM KH_2_PO_4_, and 1 mM PMSF, pH 7.4) twice and stored at −80°C until the luciferase assay was performed. Pellets were re-suspended in 300 μL lysis buffer, proteins were extracted by bead-beating with lysates maintained on ice, and extracted protein concentrations were estimated by a BCA (bicinchoninic acid) protein assay kit. Equal total protein concentrations of lysates were used while measuring the luciferase activity, using 2 μg/μL protein for a 20 μL reaction. Luciferase activity was measured through a luciferase assay kit (Promega, E1500) and a luminometer (Sirius, Titertek Berthold Detection Systems). The luciferase activities for each replicate (relative light units per second [RLU/s]) were normalized with individual controls (+AA). The relative RLU in luciferase activities between different time points in minimal media was used to estimate changes in the active translation of Gcn4 transcription factor proteins.

### Growth assays

The growth of auxotrophic strains was monitored on synthetic minimal medium (as described earlier) agar plates. Primary cultures of auxotrophic cells were washed, resuspended in 1 mL medium at an OD_600_ of 1.0, serially diluted 10× into multiple tubes, and a 5 μL drop from each 10× dilution was spotted onto minimal medium agar plates without amino acids, or with the respective (auxotroph) amino acid supplemented. Auxotrophic strains used are listed in Table S2.

### Assay for growth using conditioned supernatant

Prototrophic wild-type *S. cerevisiae* cells were grown in minimal medium with 2% glucose, without amino acids, for 24 hours at 30°C in a shaker incubator. The supernatant was harvested after 24 hours of growth and filtered using a 0.22 μm membrane. To this spent medium, only glucose (2%) and ammonium sulfate (~35 mM) were added to make “conditioned supernatant.” This was used as the medium source to test the growth of the respective auxotrophic strains (Table S2). For growth experiments, a primary culture of the specified auxotrophs was grown overnight in YPD nutrient broth, and the primary culture was collected, washed, and diluted to an OD_600_ ~0.10, and grown in the conditioned supernatant at 30°C in the shaker incubator.

### Co-cultures of private-public and public-public auxotroph goods

Conditioned spent medium (described above) was used for individual strain culture, as well as for co-cultures of combinations of different auxotrophic cells. Combinations of auxotrophic cell pairs were seeded at an OD_600_ of 0.05 each in the conditioned supernatant. In co-cultures, the starting ratios of the two auxotrophs were 50:50 (ratio), and at a starting OD_600_ of 0.1, grown in 100 mL flasks containing 20 mL conditioned supernatant. The growth of the co-cultures and their fractional compositions were determined by counting colony-forming units (CFU), as well as counting cells with reporter expression, where cells were labeled with green (mNeonGreen) or red (mCherry) fluorescent tags at an endogenous locus (Table S2).

### Cell counting

Co-cultured cells expressing a fluorescence reporter in conditioned supernatant were visualized using a microscope (Olympus BX53) with a 20× air objective. The fraction of cells expressing each reporter was then estimated by counting.

### Data representation and statistical analysis

Graphs were plotted and data analyzed using GraphPad Prism 6. Two-sample, paired, or two-tailed Student’s *t* tests were used (as indicated) to estimate statistical significance unless otherwise specified. For flux experiments, the raw intensity values for each replicate were normalized to respective controls. For mass spectrometry experiments, relative levels in spent media and conditioned spent media were compared. *P* values and *n* for corresponding experiments have been specified in figure legends.

## Supplementary Material

**Additional Files**

The following material is available online.

Supplementary information

Supplementary Worksheet

Supplementary Worksheet 1

## Figures and Tables

**Table 1:** Absolute intracellular amino acid concentrations and extracellular amino acid concentrations^a^

Amino acid	Absolute intracellular concn (μM)						Extracellular concn (μM)				
	2 h	4 h	8 h	12 h	24 h		2 h	4 h	8 h	12 h	24 h
Alanine	268.5 ± 36.2	769.0 ± 16.5	930.6 ± 48.1	954.2 ± 59.4	920.4 ±198.5		31.6 ± 1.7	65.3 ± 10.6	222.2 ± 12.0	540.3 ± 34.4	268.1 ± 11.5
Arginine	91.8 ± 3.2	82.5 ± 5.3	87.3 ± 2.0	88.1 ± 2.5	129.3 ± 3.5		10.6 ± 3.5	19.5 ± 11.0	12.9 ± 9.1	10.7 ± 11.5	37.1 ± 3.8
Asparagine	88.2 ± 18.3	180.6 ± 8.6	110.3 ± 6.5	123.1 ± 22.3	402.8 ± 49.1		39.6 ± 31.2	17.6 ± 11.4	39.2 ± 20.7	94.3 ± 9.9	92.4 ± 28.8
Aspartic acid	546.5 ± 22.4	880.1 ± 33.5	559.6 ± 28.4	518.9 ± 22.2	107.9 ± 11.9		16.3 ± 4.2	43.4 ± 3.2	47.2 ± 13.2	30.2 ± 27.8	15.5 ± 2.9
Glutamate	2,229.5 ± 205.2	2,693.0 ± 29.2	1,882.8 ± 59.6	1,650.0 ± 92.1	1,183.1 ± 153.8		266.0 ± 21.3	428.8 ± 55.8	449.1 ± 55.5	863.6 ± 184.9	914.8 ± 17.3
Glutamine	1,636.5 ± 132.6	2,911.8 ± 81.2	2,072.5 ± 54.9	2,058.9 ± 196.0	1,486.5 ± 73.7		278.5 ± 22.0	672.9 ± 54.5	972.8 ± 108.4	1,646.4 ± 125.1	902.3 ± 24.5
Histidine	152.3 ± 17.5	246.3 ± 9.6	239.0 ± 13.9	235.5 ± 20.4	214.9 ± 20.8		14.6 ± 4.2	16.0 ± 1.4	14.8 ± 11.0	10.2 ± 3.7	19.9 ± 9.9
Leucine/isoleucine	48.5 ± 5.8	103.6 ± 4.8	51.6 ± 3.9	78.9 ± 7.0	568.0 ± 91.6		28.7 ± 1.1	44.2 ± 5.1	9.5 ± 2.8	211.5 ± 12.2	183.3 ± 31.3
Lysine	192.8 ± 17.0	269.2 ± 5.4	234.8 ± 24.4	368.7 ± 28.8	296.1 ± 23.8		ND	ND	ND	ND	3.4 ± 0.2
Methionine	1.4 ± 0.5	2.8 ± 0.4	ND	ND	ND		1.0 ± 0.1	1.8 ± 0.1	1.8 ± 0.1	1.8 ± 0.1	1.9 ± 0.1
Phenylalanine	15.6 ± 2.3	28.3 ± 2.2	18.9 ± 0.8	21.2 ± 2.3	109.9 ± 23.2		15.2 ± 0.8	18.5 ± 2.4	34.3 ± 1.3	43.9 ± 2.9	25.0 ± 1.7
Proline	90.0 ± 12.2	174.4 ± 3.8	56.4 ± 1.4	53.0 ± 2.7	179.4 ± 23.8		131.4 ± 8.6	362.0 ± 14.7	373.7 ± 11.3	220.2 ± 18.1	199.1 ± 7.7
Serine	241.1 ± 41.8	489.8 ± 26.4	166.4 ± 4.6	167.2 ± 11.7	258.1 ± 57.9		22.8 ± 4.4	35.3 ± 2.7	48.3 ± 18.6	97.2 ± 20.6	106.1 ± 7.1
Threonine	151.4 ± 14.4	327.2 ± 9.2	130.4 ± 2.8	142.7 ± 13.8	395.7 ± 44.5		28.4 ± 4.2	63.2 ± 4.7	87.4 ± 7.6	172.8 ± 12.8	170.5 ± 9.7
Tryptophan	8.3 ± 0.8	15.4 ± 0.8	14.1 ± 0.6	16.3 ± 2.0	21.3 ± 1.6		6.1 ± 1.3	7.2 ± 1.5	11.6 ± 1.8	17.5 ± 2.6	10.2 ± 0.2
Tyrosine	8.2 ± 0.8	15.1 ± 1.1	28.2 ± 1.9	38.3 ± 3.3	212.9 ± 25.6		3.5 ± 0.8	3.1 ± 0.4	6.5 ± 0.6	10.6 ± 0.7	76.8 ± 3.5
Valine	143.0 ± 18.5	323.7 ± 11.9	266.6 ± 8.8	290.7 ± 17.7	274.7 ± 5.1		20.3 ± 0.9	31.4 ± 3.6	70.1 ± 6.6	152.0 ± 8.4	81.6 ± 0.9

a ND, not detected.

## Data Availability

The raw mass spectrometry data files in this study are available in the publicly accessible Indian Biological Data Centre metabolome data archive (IBDC; https://ibdc.dbt.gov.in/) at https://ibdc.dbt.gov.in/imda/study_details_url_MS/60/6/ (Project Accession IMP_100062; Study Accession IMS_100060). The normhttps://ibdc.dbt.gov.in/imda/study_details_url_MS/60/6/ https://ibdc.dbt.gov.in/imda/study_details_url_MS/60/6/https://ibdc.dbt.gov.in/imda/study_details_url_MS/60/6/https://ibdc.dbt.gov.in/imda/study_details_url_MS/60/6/ https://ibdc.dbt.gov.in/imda/study_details_url_MS/60/6/alized area under the curve data for reported metabolites in this study are provided as Excel worksheets in the supplemental material.

## Importance

Amino acids are central to a metabolic economy and are extensively exchanged between cells; yet, the scale of this economy remains unknown even in model microbes. This study establishes a quantitative blueprint of the amino acid economy in *Saccharomyces cerevisiae* by mapping production, secretion, and consumption fluxes. The findings reveal a distinction between public goods—such as alanine, which is secreted and re-consumed—and privatized resources, such as aspartate, which cells retain to sustain carbon metabolism. These pools shift across growth phases and move toward privatization during nitrogen limitation. By defining these frameworks, this study enables the rational design of stable, synthetic communities of auxotrophs.
