# Supplementary material for: The quantitative amino acid economy of yeast reveals public versus private goods and guides syntrophic community design
Source: mSystems. 2026 Aug 7;11(9):e00333-26. doi: 10.1128/msystems.00333-26 (PMC7619428; doi:10.1128/msystems.00333-26)
Supplement: Supplementary file 1 [file msystems.00333-26-s0001.docx]

**Supplementary information**

**The Quantitative Amino Acid Economy of Yeast Reveals Public versus Private Goods and Guides Syntrophic Community Design**

**Authors:** Shabbir Ahmad & Sunil Laxman*

iBRIC Institute for Stem Cell Science and Regenerative Medicine (inStem)

GKVK Post Bellary Road

Bangalore 560065

*corresponding author: [sunil@instem.res.in](mailto:sunil@instem.res.in)

**Supporting information**

**Contents**

Tables

Table S1 Plasmid used in this study

Table S2 All the strains used in this study

Table S3 Mass spectrometry Q1 and Q3 parameters for all metabolites detected

Supporting Figures

Fig. S1: Privatized amino acids – costs, demand, and metabolic uses within a cell.

Fig. S2: Growth kinetics of *S. cerevisiae* in nitrogen replete or nitrogen starved medium

Fig. S3: Growth kinetics of two *S. cerevisiae* public or private good auxotrophs

Fig. S4: Relative amounts of intracellular and extracellular amino acids in a private good auxotroph grown with exogenous supplementation

Fig. S5: Relative amounts of intracellular and extracellular amino acids in a public good auxotroph grown with exogenous supplementation

Fig. S6: Growth of auxotrophic strains of public good or private good amino acids

Supporting worksheets and raw datasets

Supplementary Worksheet – contains quantitative standards for all amino acids measured using the LC/MS/MS methods in this study provided as an excel sheet

Mass spectrometry raw data – contains data from each replicate for MS/MS based quantification of intracellular or extracellular amino acids provided as an excel sheet

**Table S1**. Plasmid used in this study

| **Plasmid** | **Description** | **Selection marker** | **Sources** |
| --- | --- | --- | --- |
| SL80 | p417-TEF1 plasmid (CEN.ARS) | G418 | Walvekar et al., 2020 |
| SL147 | Luciferase reporter gene in SL80 | G418 | Walvekar et al., 2020 |
| SL 148 | Gcn4 uORFs (500 bp upstream of ATG start codon) fused to codons encoding first 55 aa of Gcn4 followed by a complete luciferase sequence, in the SL80 plasmid. | G418 | Walvekar et al., 2020 |

**Table S2**. All the strains used in this study

| **Strains** | **Description** | **Selection marker** | **Sources** |
| --- | --- | --- | --- |
| CEN.PK | prototroph | Mat a | Van Dijken et al., 2000 |
| SL65 | *lys1Δ* | G418 | This study |
| SL1109 | *his3Δ* | G418 | This study |
| SL1111 | *met*6*Δ* | G418 | This study |
| SL1115 | *trp*5*Δ* | G418 | This study |
| SL1160 | *bat1/bat2Δ* | G418, hyg | This study |
| SL1165 | *bat1Δ/leu2Δ* | G418, hyg | This study |
| SL1326 | *lys1Δ*/ mCherry | G418, NAT | This study |
| SL1164 | *met6Δ*/ mCherry | G418, hyg | This study |
| SL1163 | *trp5Δ*/mCherry | G418, hyg | This study |
| SL1161 | *his3Δ*/mCherry | G418, hyg | This study |
| SL1325 | *bat1Δ/bat2Δ* /mNeonGreen | G418, hyg, NAT | This study |

**Table S3**. Mass spectrometry Q1 and Q3 parameters for all metabolites detected

| **Name** | **Q1(Parent)** | **Q3 (Product)** | **Collision energy (CE)** |
| --- | --- | --- | --- |
| His | 156.0 | 110.0 | 12 |
| Phe | 166.0 | 103.0 | 25 |
| Tyr | 182.2 | 136.0 | 14 |
| Trp | 205.0 | 146.0 | 15 |
| Ala | 90.0 | 44.0 | 11 |
| Ser | 106.0 | 60.0 | 13 |
| Val | 118.0 | 55.0 | 15 |
| Leu/IIe | 132.0 | 86.0 | 11 |
| Glu | 148.1 | 84.1 | 16 |
| Gln | 147.0 | 84.0 | 16 |
| Asp | 134.1 | 74.0 | 15 |
| Asn | 133.0 | 74.0 | 15 |
| Thr | 120.0 | 57.0 | 30 |
| Arg | 175.2 | 60.0 | 14 |
| Pro | 116.0 | 70.0 | 11 |
| Lys | 147.0 | 84.0 | 16 |
| Met | 150.1 | 56.0 | 15 |
| **^15^N incorporation from ammonium sulfate** | | | |
| Name | **Q1(Parent)** | **Q3 (Product)** | **CE** |
| Asp | 134.1 | 74.0 | 15 |
| ^15^N_Asn+Asp (m+1) | 135.1 | 75.0 | 14 |
| Arg | 175.2 | 60.0 | 14 |
| ^15^N_Arg (m+1) | 176.2 | 61.0 | 15 |
| Met | 150.1 | 56.0 | 15 |
| ^15^N_Met (m+1) | 151.1 | 57.0 | 15 |
| Gln | 147.0 | 84.0 | 16 |
| ^15^N_Glu+Gln (m+1) | 149.1 | 85.1 | 15 |
| Lys | 147.0 | 84.1 | 15 |
| ^15^N_Lys (m+1) | 148.0 | 85.1 | 15 |
| His | 156.0 | 110.0 | 12 |
| ^15^N_His (m+1) | 157.0 | 111.0 | 12 |
| Ser | 106.0 | 60.0 | 13 |
| ^15^N_Ser (m+1) | 107.0 | 61.0 | 13 |
| Thr | 120.0 | 57.0 | 30 |
| ^15^N_Thr (m+1) | 121.0 | 57.0 | 30 |
| Pro | 116.0 | 70.0 | 11 |
| ^15^N_Pro (m+1) | 117.0 | 71.0 | 11 |
| Val | 118.0 | 72.0 | 15 |
| ^15^N_Val (m+1) | 119.0 | 55.0 | 15 |
| Leu/IIe | 132.0 | 86.0 | 11 |
| ^15^N_Leu/Ile (m+1) | 133.0 | 87.0 | 11 |
| Phe | 166.0 | 103.0 | 25 |
| ^15^N_Phe (m+1) | 167.0 | 103.0 | 25 |
| Trp | 205.0 | 146.0 | 15 |
| ^15^N_Trp (m+1) | 206.0 | 147.0 | 15 |
| Tyr | 182.2 | 136.0 | 14 |
| ^15^N_Tyr (m+1) | 183.2 | 137.0 | 14 |
| **Stable isotopes ^13^C_4_-Asp** $\boldsymbol{\to}$ **incorporation into metabolites** | | | |
| **Compound** | **Q1(Parent)** | **(Product)** | **CE** |
| G-3-P | 169.0 | 97.0 | -20 |
| ^13^C_G-3-P (m+3) | 172.0 | 97.0 | -20 |
| PEP | 167.0 | 79.0 | -12 |
| ^13^C_PEP (m+3) | 170.0 | 79.0 | -12 |
| 3-PG | 185.0 | 97.0 | -20 |
| ^13^C_3-PG (m+3) | 188.0 | 97.0 | -20 |
| R-5-P | 229.0 | 97.0 | -20 |
| ^13^C_R-5-P (m+3) | 232.0 | 97.0 | -20 |
| S7P | 289.0 | 97.0 | -20 |
| ^13^C_S7P (m+3) | 292.0 | 97.0 | -20 |
| GDP-Man | 605.8 | 384.9 | -37 |
| ^13^C GDP-Man (m+3) | 608.8 | 384.9 | -37 |
| UDP-Glc | 665.0 | 323.00 | -25 |
| ^13^C UDP-Glc (m+3) | 668.0 | 323.00 | -25 |
| **OBHA derivatized TCA cycle intermediates** | | | |
| **Metabolite** | **Q1(Parent)** | **Q3(Product)** | **CE** |
| Pyruvate | 299.0 | 181.0 | 15 |
| ^13^C_ Pyruvate (m+3) | 302.0 | 181.0 | 15 |
| Citric acid | 508.0 | 92.2 | 7 |
| ^13^C_ Citric acid (m+4) | 512.0 | 92.2 | 7 |
| α-KGA | 462.0 | 339.0 | 11 |
| ^13^C_ α-KGA (m+4) | 466.0 | 343.0 | 11 |
| Succinate | 329.0 | 91.1 | 34 |
| ^13^C_Succinate (m+2) | 331.0 | 91.1 | 34 |
| Malate | 345.0 | 91.2 | 33 |
| Malate (m+4) | 349.0 | 91.2 | 33 |
| **d4-alanine conversion to pyruvate** | | | |
| **Compound** | **Q1 (Parent)** | **Q3(Product)** | **CE** |
| Pyruvate | 299.0 | 181.0 | 15 |
| D4-Pyruvate (m+3) | 302.0 | 181.0 | 15 |

**Supplementary Fig. S1:**

**Supplementary Fig. S1: Privatized amino acids – costs, demand, and metabolic uses within a cell.** The table (top) indicates the biosynthetic cost (per molecule) of each of the privatized amino acid, as well as the total demand coming from metabolic and protein synthesis uses (from PMID 39179593). The illustration shows the diverse, dynamic metabolic uses of each of the privatized amino acids. Aspartate is utilized in carbon metabolism, amino acid biosynthesis and nucleotide biosynthesis, in addition to its use in protein synthesis. Arginine is critical for polyamine biosynthesis, as well as is a major proportion of amino acids in ribosomal proteins. Lysine also is used extensively in ribosomes, but has minor metabolic demands (limited to acetyl-CoA biosynthesis). Methionine is the primary precursor for s-adenosyl methionine, the major methyl donor for all biological methylation. Histidine has critical uses as a cellular buffer, supports the folate cycle and balances glutamate synthesis.

**Supplementary Fig. S2**: Growth kinetics of *S. cerevisiae* in standard minimal medium (Glucose 110mM ammonium sulfate 35mM) or in ammonium sulfate limited (350μM) medium over 24 hours.

**Supplementary Fig. S3: Growth of auxotrophic strains of public good or private good amino acids.** Multiple auxotrophic strains were generated in complete amino acid medium, and the extent of essentiality of the auxotrophy on the indicated strains were confirmed by growing these cells in synthetic minimal medium without any amino acid supplemented. Shown are growth assays (by serial dilution of cells on solid medium) of wild type cells, compared to auxotrophic strains of public or private good amino acids in minimal medium, and indicate that all these strains were strong auxotrophs incapable of growing without exogenously supplied amino acids.

**Supplementary Fig. S4:** Growth curves of prototrophic *S. cerevisiae*, and private (*lys1Δ*) and public good (*trp5Δ*) auxotrophs, with and without supplemented lysine or tryptophan respectively over 24 hours. N=3, mean±SD

**Supplementary Fig. S5:** Bar plots indicating the relative amounts intracellular and extracellular amino acids present in prototrophic cells, or in a private good auxotroph (*lys1Δ*) supplemented with lysine, at 12 hours of growth. Amino acids were quantitatively detected by LC-MS/MS as described earlier. n=3 (biological replicates). *p<0.05, **p<0.01, ***p<0.001 (Student’s t-test). Note: since lysine was supplemented in excess in the medium for *lys1Δ* growth, the amounts of that amino acid extremely high and so are not depicted on the respective graph.

**Supplementary Fig. S6:** Bar plots indicating the relative amounts of intracellular and extracellular amino acids in a public good auxotroph (*trp5Δ*) supplemented with tryptophan, at 12 hours of growth. Amino acids were quantitatively detected by LC-MS/MS as described earlier. n=3 (biological replicates). *p<0.05, **p<0.01, ***p<0.001 (Student’s t-test). Note: since tryptophan was supplemented in excess in the medium for *trp5Δ* growth, the amounts of that amino acid extremely high and so are not depicted on the respective graph.
